# *Allium mongolicum* Regel-Mediated Rumen Microbiota Intervention Modulates Hepatic Metabolome to Reduce 4-Alkyl Branched-Chain Fatty Acids in Lamb *Longissimus Thoracis* Muscle

**DOI:** 10.3390/foods15101617

**Published:** 2026-05-07

**Authors:** Xiaoyuan Wang, Xinyi Liu, Guoli Han, Khas Erdene, Chen Bai, Qina Cao, Yankai Zheng, Lahan Hai, Changjin Ao

**Affiliations:** Key Laboratory of Animal Nutrition and Feed Science, College of Animal Science, Inner Mongolia Agricultural University, Hohhot 010018, China

**Keywords:** *Allium* plants, gamey flavor, rumen fluid transplantation, metagenomics, gut-liver axis

## Abstract

Deposition of three key 4-alkyl branched-chain fatty acids (**KBCFA**), including 4-methyloctanoic acid (**MOA**), 4-ethyloctanoic acid (**EOA**), and 4-methylnonanoic acid (**MNA**), causes the gamey flavor in sheep meat. This study integrated metagenomics and metabolomics to evaluate how *Allium mongolicum* Regel (**AMR**) supplementation (15 g/d) and rumen fluid transplantation (**RFT**) modulate rumen microbiota and hepatic metabolism to reduce KBCFA in lamb *longissimus thoracis* muscle. The experiment consisted of two phases. In Phase I, twelve 3-month-old male Dorper × Small Tailed Han sheep (25 ± 1 kg) were selected as the rumen donor group. These sheep were supplemented with 15 g/d/head of AMR powder in their basal diet until the end of the experiment. In Phase II, thirty 3-month-old male Dorper × Small Tailed Han sheep (23 ± 2 kg) were randomly assigned to one of three groups (*n* = 10 per group): the control group (STG), which was fed the basal diet and received a physiological saline transplant; the AMR group, which was fed the basal diet supplemented with 15 g/d/head of AMR powder and received a physiological saline transplant; and the rumen fluid transplant group (RTG), which was fed the basal diet and received a rumen fluid transplant from the donor group. Compared to the STG, results showed that the MOA, EOA, and MNA in the AMG decreased by 64.51%, 54.72%, and 49.34%, respectively. Similarly, the MOA, EOA, and MNA in the RTG were reduced by 63.13%, 56.17%, and 49.60%, respectively (*p* < 0.001). For the rumen metagenome, AMR enriched the genus *Prevotella*, while RFT increased *Butyrivibrio*. Hepatic metabolomics revealed a distinct shift where AMR elevated amino acid derivatives and RFT enhanced carnitine-related metabolites. These alterations indicate a potential metabolic shift associated with amino acid metabolism and mitochondrial β-oxidation, rather than lipid elongation. We postulate that this coordinated regulation across the rumen–liver–muscle axis may alter the availability of lipogenic precursors for KBCFA synthesis, ultimately contributing to improved meat flavor.

## 1. Introduction

Sheep meat is an excellent dietary source of highly bioavailable proteins, indispensable amino acids, and polyunsaturated fatty acids [1], contributing significantly to global food security [2]. However, unlike beef or pork, lamb is characterized by a distinctive “gamey” flavor [3], which significantly restricts consumer acceptance and challenges the livestock industry [4]. The primary contributors to gamey flavor, specifically, three key branched-chain fatty acids (**KBCFA**), namely 4-ethyloctanoic acid (**EOA**), 4-methylnonanoic acid (**MNA**), alongside 4-methyloctanoic acid (**MOA**) [5]. The accumulation of these compounds is closely linked to dietary composition; for instance, high-energy diets exacerbate KBCFA deposition, thereby intensifying gamey flavor attributes. This indicates that dietary intervention represents a potential strategy for modulating the formation of specific mutton flavor compounds [6].

The medicinal plant *Allium mongolicum* Regel (**AMR**), which acts as a functional species within the *Allium* genus, abounds in specific bioactive components. Its primary active constituents include specific flavonoids, such as quercetin and kaempferol glycosides and 7-O-5,4′-dimethoxy-3′-hydroxyflavonol, alongside polysaccharides rich in D-glucose and L-rhamnose [7]. These bioactive constituents possess notable antioxidant and gut-protective properties. Active compounds, including alkaloids, organic acids, and specific flavonoids such as quercetin and chrysoeriol, can suppress bacterial metabolism by disrupting cell membranes and inhibiting protein synthesis. This targeted antimicrobial action modulates the rumen microbiota to optimize fermentation, thereby increasing the absorption of beneficial nutrients and antioxidants. The subsequent deposition of these molecules into muscle tissue, where they not only reduce KBCFA concentration, yellowness, and hue angle in lamb, but also prolong its storage life [8]. Previous studies demonstrate that 3.4 g/d of AMR supplementation alters ruminal microbiota, specifically enhancing *Tenericutes* and *Mollicutes*, and mitigates gamey flavor by reducing KBCFA content by approximately 41% to 75% in muscle [9]. Zhao et al. [10] reported that supplementation with 2.8 g/d of ethanolic extract from AMR reduced the levels of MOA as well as MNA accumulated within the liver by approximately 33% and 50%, respectively, while reducing key rumen genera such as *Prevotella* and *Succiniclasticum*. However, while a correlation between AMR supplementation, microbial shifts, and reduced KBCFA levels has been established, it is unclear whether the reduction in KBCFA is driven by the direct absorption of the plant’s bioactive compounds or mediated primarily through induced microbial changes. In this context, rumen fluid transplantation (**RFT**) was employed to further investigate the contribution of the AMR-modulated ruminal environment. By transplanting rumen fluid from AMR-adapted donors into naive recipients, the recipients acquire the AMR-altered microbiota and associated ruminal metabolites without directly and continuously ingesting AMR. This approach minimizes the primary direct absorption of the plant’s intact biomass and bioactive components. RFT has been shown to regulate host physiological status through microbial transfer and influence product quality by modulating lipid metabolism pathways [11,12]. Therefore, comparing direct dietary AMR supplementation with AMR-induced RFT offers a valuable experimental framework to better understand the extent to which the AMR-modulated ruminal ecosystem contributes to meat flavor regulation, as opposed to direct dietary intake alone.

We hypothesized that the AMR-modulated ruminal environment reduces the accumulation of KBCFA within the *longissimus thoracis* (**LT**) muscle of sheep by modulating both rumen microbiota and hepatic metabolic processes. Based on this hypothesis, if the RFT recipients exhibit a comparable reduction in KBCFA to the direct AMR-supplemented group, it would suggest that the transferred ruminal ecosystem significantly contributes to this effect. To comprehensively investigate this, we integrated rumen metagenomics with hepatic metabolomics to evaluate the relationships among AMR supplementation, microbial community structure, hepatic metabolic pathways, and KBCFA deposition. By directly comparing AMR supplementation with AMR-based RFT, the present work delivers fresh mechanistic perspectives concerning the regulation of KBCFA metabolism along the rumen microbiota–liver–muscle axis, providing insights into the relative contributions of direct dietary intake versus the AMR-modulated ruminal environment upon meat flavor.

## 2. Materials and Methods

### 2.1. Animal Ethics and Welfare

All experimental procedures received approval from Inner Mongolia Agricultural University’s Animal Welfare Committee… in strict compliance with the guidelines for ethical animal care issued by the Ministry of Science and Technology of China.

### 2.2. Preparation of the AMR Supplement

Wild-growing AMR was collected within the region of Bayanhot (Alxa Left Banner, Inner Mongolia, China) across its primary growing phase (between July and September). Fresh AMR plant material received consistent drying treatment at 65 °C to retain active component potency. Following dehydration, samples were processed via DFT-300 grinding equipment (purchased from Shanghai Xinnuo Instrument Co., Ltd., Shanghai, China), followed by screening using 80-mesh filtration to obtain the final AMR supplement. This ground material was then enclosed in airtight bags alongside being preserved at 4 °C until lamb feeding. The nutritional composition of AMR on a dry matter (**DM**) basis was as follows: crude protein (**CP**, 32.55%), calcium (**Ca**, 0.97%), acid detergent fiber (**ADF**, 15.87%), phosphorus (**P**, 0.58%), neutral detergent fiber (**NDF**, 17.55%), alongside ether extract (**EE**, 6.25%) [8].

### 2.3. Study Design and Husbandry Practices

The in vivo experiment was conducted within the Fuchuan Breeding Sheep facility located in Bayannur, Inner Mongolia, China, from January to June 2025. The experiment consisted of two phases lasting a total of 135 days: Phase I aimed to establish a donor model for rumen fluid, whereas the RFT trial was carried out during Phase II.

In Phase I, twelve 3-month-old male crossbred sheep (Small-tail Han × Dorper) with an initial body weight of 25 ± 1 kg were assigned to serve as rumen fluid donors. To ensure the continuous effectiveness of the donor rumen fluid, dietary supplementation with AMR continued throughout the entire experimental duration (135 days). In Phase II, thirty 3-month-old male crossbred sheep (Small-tail Han × Dorper) with an initial body weight of 23 ± 2 kg were randomly assigned into three distinct experimental cohorts (10 animals/group). Animals in the control cohort were fed the basal diet alongside orally administered saline (**STG**, *n* = 10). Sheep assigned to the AMR intervention cohort consumed the basal diet supplemented with 15 g/d of AMR per individual, coupled with an oral saline dose (**AMG**, *n* = 10). Conversely, the RFT cohort received the basal diet in conjunction with an oral dose of rumen fluid collected from the Phase I donor sheep (**RTG**, *n* = 10). The Phase II started on day 60 of the trial and spanned a total of 75 days, consisting of an adaptation period of 15 days, which was followed by a 60-day main feeding trial. At the start of day 16 during Phase II, AMR supplementation was initiated for the AMG, whereas RFT was implemented for the RTG. The administration level of AMR was selected according to prior research trials [13].

Lambs were housed in south-facing pens to ensure a minimum of 10 h of daily natural light, with ad libitum access to water. Ambient temperature and humidity were controlled at 20 ± 5 °C and 50 ± 10%, respectively. The identical basal ration was provided to all subjects two times a day (at 07:00 and 17:00), and leftover feed was documented every morning at 06:30. The 15 g/d AMR dose was blended with the initial third (0.8 kg) of the morning ration; the remaining feed was provided only after complete consumption of this mixture (Table 1).

**Figure 1:**
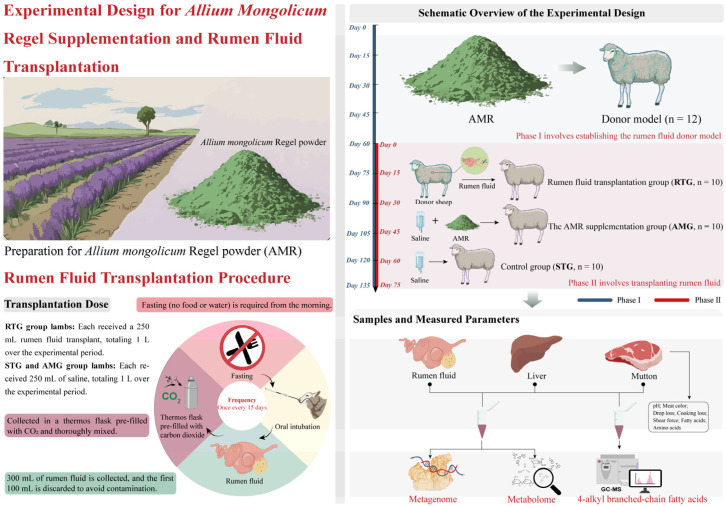
A diagram of the study design that included *Allium mongolicum* Regel (AMR) supplementation and rumen fluid transplantation. The study workflow includes the preparation of AMR powder and a two-phase animal experiment. Phase I involved establishing the donor model (*n* = 12) using AMR. Phase II assigned the recipient sheep to three groups (10 animals per group): the STG (control group, receiving saline), AMG (AMR supplementation group, receiving AMR and saline), and RTG (rumen fluid transplantation group, receiving donor rumen fluid). The transplantation procedure involved collecting rumen fluid in CO_2_-pre-filled flasks and administering 200 mL via oral intubation every 15 days after fasting. Finally, biological samples including rumen fluid, liver tissue, and mutton were collected to perform metagenomic sequencing, metabolomic analysis, and 4-alkyl branched-chain fatty acid levels measured by GC-MS.

Lambs in Phase II were fasted from feed and water on the morning of each transplantation day. Rumen fluid collection and transplantation were strictly performed in accordance with the protocol established by Liu et al. [14]. In summary, rumen fluid was collected from each of the twelve donor lambs in Phase I via an oral stomach tube equipped with a metal filter. The first 100 mL drawn was removed to avoid saliva contamination. Thereafter, a 250 mL sample of rumen fluid was taken from each donor and immediately placed into CO_2_-flushed bottles to maintain anaerobic conditions. The fluid from all donors was thoroughly mixed. Each RTG animal was administered 250 mL of the donor ruminal fluid via oral gavage, while STG and AMG animals received 250 mL of saline solution. The transplantation procedure was conducted every 15 days, totaling four administrations. Consequently, during Phase II, STG and AMG animals each received a total of 1 L of saline, while each RTG lamb received 1 L of ruminal fluid from donors. Figure 1 depicts the experimental protocol and transplantation methodology.

### 2.4. Sample Collection

At the end of the experimental period, all lambs were slaughtered following standard commercial procedures. Ruminal fluid samples were immediately collected. Approximately 150 mL of ruminal fluid was filtered through four layers of sterile cheesecloth, kept at −20 °C for fermentation parameter analysis. Another 150 mL sample was taken and rapidly frozen at −80 °C for metagenomic sequencing. The LT muscle (approximate 150 g) was dissected from the region excised from the area between the 12th and 13th ribs, then instantly flash-frozen at −80 °C pending KBCFA analysis. Liver samples were obtained after capsule removal and stored at −80 °C for metabolomic assessment.

### 2.5. Experimental Sampling Strategy

A sub-cohort of six samples per group were selected for hepatic metabolomics and rumen metagenomics. To minimize subjective selection bias, these samples were chosen objectively based on their ruminal bacterial profiles evaluated using principal coordinate analysis (PCoA) of 16S rRNA sequencing data from a concurrent study [15]. Specifically, the six individuals whose microbial profiles were located closest to the centroid of their respective group clusters in the PCoA were selected.

### 2.6. Analysis of Feed Samples

The nutritional composition of feed samples was analyzed according to standard AOAC methods [16]. Specifically, DM, CP, EE, Ca, P, NDF and ADF were analyzed using methods 934.15, 990.03, 920.39, 968.05, 975.16, 2002.04, and 973.19, respectively.

### 2.7. Analysis of Rumen Fermentation

A portable pH meter was employed to determine ruminal fluid pH values immediately post-collection (PH-3C, Shanghai Osterol Industrial Co., Shanghai, China) calibrated with standard buffers. Following pH measurement, the rumen fluid was centrifuged at 4000× *g* for 10 min (5425 R, Eppendorf China, Shanghai, China), and the supernatant was stored at −20 °C for the measurement of ammonia nitrogen (**NH_3_-N**) and volatile fatty acids (**VFAs**). The NH_3_-N levels were measured by the indophenol colorimetric method of Chaney and Marbach [17]. VFA profiles were analyzed using a gas chromatography system (GC-2014, Shimadzu, Kyoto, Japan) fitted with a flame ionization detector (**FID**). Separation was achieved using an InertCap 35 capillary column (60 m × 0.25 mm i.d. × 0.50 μm film thickness; Shimadzu, Japan). GC conditions were as follows: the injector was at 220 °C, the detector at 250 °C, and the column oven was kept at 180 °C. High-purity nitrogen was used as the carrier gas at 1.74 mL/min with a linear velocity of 32.6 cm/s. The split ratio was 40:1, and makeup gas (nitrogen), hydrogen, and air flow rates were 3, 55, and 40 mL/min, respectively. Data were analyzed using the method of Guo et al. [18].

### 2.8. Determination of KBCFA in LT Muscle

4-alkyl branched-chain fatty acids were quantified using a Trace 1310 ISQ GC-MS system (Thermo Fisher Scientific, Waltham, MA, USA). To enhance analytical volatility and stability, KBCFA were converted to methyl esters following the procedure described by Del Bianco et al. [19]. In brief, 2 mL of 2.0 mol/L NaOH was added to hydrolyze 0.5 g of dried muscle tissue in methanol at 110 °C for 2 h. After cooling to room temperature, the samples were mixed with 2 mL of 14% BF_3_-methanol solution and heated at 80 °C for 15 min. Following this, double extraction was performed using diethyl ether. The pooled organic layers were evaporated to approximately 1 mL under a gentle nitrogen stream. The extraction and quantification procedures were adapted from the method described by Liu et al. [8]. Crucially, quantification was performed using an internal standard method. Undecanoic acid (C11:0) was added as an internal standard prior to the extraction process to account for matrix effects and potential extraction losses.

The LOD values were 0.15 mg/kg (MOA), 0.11 mg/kg (EOA), and 0.11 mg/kg (MNA). The LOQ was three times the LOD. Precision was confirmed by analyzing quality control samples, with relative standard deviations (**RSD**) < 10%. Futhermore, the recovery rate of the internal standard ranged from 93% to 120%.

MS was conducted in EI mode at 70 eV. A 1 μL sample was injected at a 10:1 split ratio into a DB-WAX capillary column (30 m × 0.25 mm i.d. × 0.5 μm film thickness; Agilent Technologies, Santa Clara, CA, USA). The injector temperature was 250 °C. The column oven temperature began at 75 °C (held for 2 min), rose to 230 °C at 10 °C/min, and was held for 7 min. Helium (99.999%) served as the carrier gas at 0.65 mL/min. The MS transfer line was set to 250 °C. Target ions at m/z 99 and 113 were monitored in SIM mode for quantification. KBCFA concentrations were calculated using the following formula:

(1) $$W\;=\;\frac{\left(C\;-\;C_{0}\right)\;\times \;V\;\times \;N}{m}\times \frac{1}{1000}$$

W denotes the KBCFA content in the sample (mg/kg); expressed on a dry-weight basis; *C* signifies the KBCFA concentration in the sample solution (mg/L); *C*_0_ stands for the concentration of KBCFA in the blank control (mg/L); *V* denotes the volume of the aliquot (mL); *N* signifies the dilution factor; m stands for the mass of the sample (g).

### 2.9. Rumen Metagenome

#### 2.9.1. DNA Extraction and Sequencing

Genomic DNA from the microbial community was extracted from rumen fluid using the MagBeads FastDNA Kit for Soil (MP Biomedicals, Irvine, CA, USA) according to the manufacturer’s protocol. DNA concentration and integrity were measured with a Qubit 4 Fluorometer using the 1X dsDNA HS Assay Kit (Invitrogen, Carlsbad, CA, USA) and 1% agarose gel electrophoresis. Libraries with a 400 bp insert size were constructed with the Illumina TruSeq Nano DNA LT Library Preparation Kit. Metagenomic shotgun sequencing was conducted on the Illumina NovaSeq platform (Personal Biotechnology Co., Ltd., Shanghai, China) to produce 150 bp paired-end (PE150) reads. A total of 18 libraries were sequenced, yielding approximately 5.5 to 8.6 Gbp of raw data per sample.

#### 2.9.2. Metagenomic Data Analysis

Initial processing of raw paired-end reads was conducted via fastp (version 0.23.2) using a 5 bp sliding-window filter [20]. Sequences were truncated when the average quality score dropped below Q20, and reads shorter than 50 bp or those containing ambiguous bases (N) were strictly discarded to ensure high-quality data. To obtain high-resolution taxonomic profiles for subsequent correlation with metabolomic data, the clean reads were directly subjected to taxonomic classification using Kraken2 (version v2.0.8-beta) [21]. A rigorous confidence threshold of 0.5 was applied against a customized integrated database, which comprised the Genome Taxonomy Database (GTDB) for bacteria and archaea, NCBI-nt for eukaryotes, and the Reference Viral DataBase (RVDB) for viruses. Subsequently, Bracken (version v2.8) was employed to accurately re-estimate the relative abundances at the species level based on the Kraken2 output. Finally, for downstream statistical frameworks, differential abundance comparisons of the taxonomic profiles between groups were systematically evaluated using the metagenomeSeq method. *p*-values were adjusted for multiple testing using the Benjamini–Hochberg False Discovery Rate (FDR) approach, with statistical significance rigorously defined as an adjusted *p*-value (adj.*p*) < 0.05.

#### 2.9.3. Statistical Analysis and Bioinformatics

β-diversity was assessed using Bray–Curtis dissimilarity for both PCoA and Non-metric Multidimensional Scaling to examine microbial community structure [22,23]. The raw sequence data from this study have been deposited in the NCBI Sequence Read Archive under accession number SRP616787.

### 2.10. Hepatic Metabolomics

#### 2.10.1. Preparation of Samples and Extraction of Metabolites

Liver tissue samples (50 mg) underwent homogenization in a tissue grinder with 200 μL of ice-cold water and two steel beads (TL-48R, Shanghai Jingxin Industry, Shanghai, China) at 55 Hz for 60 s. Metabolites were extracted by adding 800 μL methanol:acetonitrile (1:1, *v*/*v*; Sinopharm Chemical Reagent Co., Ltd, Shanghai, China), followed by a 30 min ultrasonication step (SCQ-1800F, Sonic Ultrasonic Instrument, Shanghai, China). Protein precipitation was achieved by being incubated at −20 °C for 30 min, then centrifuged at 12,000× *g* (10 min, 4 °C; 5425 R, Eppendorf China, Shanghai, China). A 800 μL aliquot of the supernatant was collected and dried under vacuum. The residues were then reconstituted in 150 μL of 50% methanol spiked with 5 ppm 2-chlorophenylalanine as an internal standard (Sinopharm Chemical Reagent Co., Ltd, Shanghai, China). After vortexing for 30 s, the solution was filtered through a 0.22 μm membrane into LC vials. QC samples were prepared by pooling 10–20 μL aliquots from each filtrate to assess instrument stability and method reproducibility.

#### 2.10.2. UPLC-MS/MS Conditions

A Waters ACQUITY UPLC HSS T3 column (2.1 × 100 mm, 1.8 μm; Waters China, Shanghai, China) at 40 °C was used for chromatographic separation with an autosampler at 8 °C. A 2 μL sample was injected. Mobile phases consisted of 0.1% aqueous formic acid (A) and 0.1% formic acid in acetonitrile (B) at a flow rate of 0.4 mL/min. The gradient was set as follows: 0–1 min, 5% B; 1–7 min, linear increase to 95% B; maintained at 95% B for 7–8 min; reverting to 5% B from 8 to 8.1 min and equilibrating at 5% for 9.1–12 min. An Orbitrap Exploris 120 (Thermo Fisher Scientific, Waltham, MA, USA) was used to collect MS data in both positive and negative ionization modes. Spray voltages were 3.5 kV (positive) and −3.0 kV (negative), with a capillary temperature of 325 °C. Sheath and auxiliary gases were set at 40 and 15 arbitrary units, respectively.

#### 2.10.3. Data Processing and Statistical Analysis

Compound Discoverer 3.3 software (Thermo Fisher Scientific) was used to process raw LC–MS data for peak detection, alignment, and retention time correction. Features deemed background noise or low quality were excluded based on peak scores. Those appearing in fewer than 50% of QC samples were removed. Missing data points were imputed using the Fill Gaps algorithm. Normalization was performed based on the Total Ion Current for individual samples. Identification of metabolites relied on matching MS1/MS2 spectral data with a self-built database (PSNGM database) and public databases (mzCloud, LIPID MAPS, HMDB, MoNA, and NIST 2020). The parameters included an MS1 mass tolerance of 15 ppm and an MS2 match factor above 50. Statistical analyses were conducted using R software (V.4.3.0, UoA, Auckland, New Zealand). The PMCMRplus package facilitated differential abundance analysis. To manage the False Discovery Rate (FDR), *p*-values underwent Benjamini–Hochberg adjustment. Metabolites exhibiting an FDR < 0.1 were deemed significantly different. Visualization (heatmaps and clustering) was performed using the pheatmap package. Pathway enrichment via KEGG was executed with clusterProfiler (v4.6.0), defining significance at *p* < 0.05 and an FDR < 0.1.

For linear discriminant analysis effect size (**LEfSe**), prior to the analysis, the taxonomic data were normalized to a total sum of 1,000,000 per sample. To minimize background noise and reduce the false positive rate associated with high-dimensional data, strict abundance filtering was applied, excluding rare taxa with a relative abundance of less than 0.1% (proportion < 0.001). The LEfSe analysis sequentially utilized the Kruskal–Wallis rank-sum test to detect features with significant differential abundance among treatments, followed by the pairwise Wilcoxon rank-sum test to assess biological consistency. Finally, linear discriminant analysis (LDA) was used to estimate the effect size of each differentially abundant taxon, with an LDA score threshold set at >2.0.

### 2.11. Statistical Analysis

Data for KBCFA concentrations and rumen fermentation parameters were analyzed using a One-way Analysis of Variance (ANOVA) with the GLM module in SAS 9.2 (SAS Institute Inc., Cary, NC, USA). Prior to the parametric analysis, the assumption of normality was evaluated using the Shapiro–Wilk test. Variables that failed to meet the normal distribution assumption were subjected to log-transformation before proceeding with ANOVA. Treatment was included as a fixed effect in the statistical model. Data are presented as means ± standard error of the mean (**SEM**). When a significant treatment effect was detected, post hoc pairwise comparisons were performed using Tukey’s honest significant difference test. Rumen fermentation data were visualized using GraphPad Prism 10 (GraphPad Software Inc., La Jolla, CA, USA) * and ** indicate *p* < 0.05 and *p* < 0.01, respectively, and “ns” denotes no significant difference.

## 3. Results

### 3.1. Rumen Fermentation Parameters

**Figure 2:**
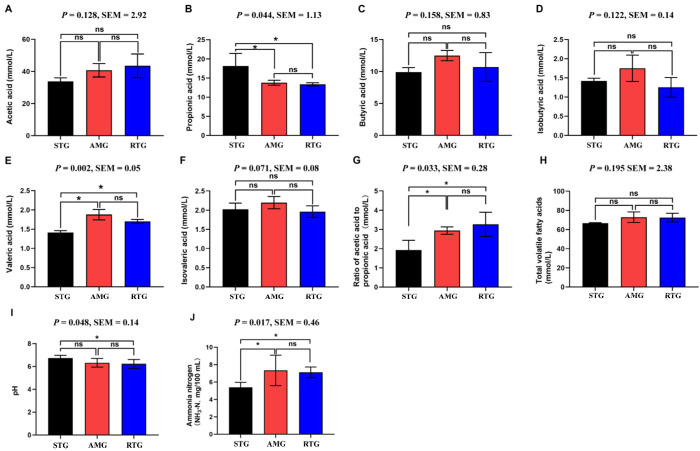
Rumen fermentation. ((**A**), Acetate; (**B**), Propionate; (**C**), Butyrate; (**D**), Isobutyrate; (**E**), Valerate; (**F**), Isovalerate; (**G**), Acetate:propionate ratio; (**H**), Total volatile fatty acids; (**I**), pH; (**J**), ammonia nitrogen). STG: lambs given a basal diet and 1 L saline in total; AMG: lambs given a basal diet with 15 g/d per lamb of *Allium mongolicum* Regel powder and 1 L saline in total; RTG: the lamb fed with a basal diet and received total of 1 L rumen fluid from donor sheep supplemented with 15 g/d of *Allium mongolicum* Regel; where * indicates *p* < 0.05, and “ns” denotes no significant difference.

Propionic acid levels in both the AMG and RTG were significantly lower than in the STG (Figure 2; *p* < 0.05), with reductions of 23.51% and 26.05%, respectively. However, the AMG and RTG did not differ significantly (*p* > 0.05). Conversely, the acetic acid to propionic acid ratio was notably increased in both the AMG and RTG compared to the STG (*p* < 0.05), showing increases of 52.33% and 69.43%, respectively. With respect to pH, the RTG demonstrated a significant decrease of 7.58% relative to the STG (*p* < 0.05), while pH levels were similar between the AMG and RTG treatments. For NH3-N, the AMG and RTG showed significantly higher levels than the STG (*p* < 0.05), with increases of 36.18% and 32.10%, respectively.

### 3.2. Levels of KBCFA Within the LT Muscle

As observed in our parallel study [24], the concentration of KBCFA is shown in Table 2. The AMG and RTG exhibited significant reductions in KBCFA concentrations (*p* < 0.001). Specifically, the AMG demonstrated decreases of 64.51% in MOA, 54.72% in EOA, and 49.34% in MNA concentrations relative to the STG (*p* < 0.001). Likewise, the RTG exhibited significant reductions of 63.13% in MOA, 56.17% in EOA, and 49.60% in MNA (*p* < 0.001).

### 3.3. Sequencing Data Summary and Assembly

A total of 793,357,260 raw reads were generated across all samples, corresponding to approximately 119.78 Gb of sequencing data, with a mean of 6.7 Gb per sample. After quality control, 772,931,190 clean reads remained, retaining 97.43% of the initial raw data. Host sequence removal (*Ovis aries*) resulted in 772,570,022 high-quality non-host reads, representing 99.95% of the clean reads. Subsequent assembly of these high-quality reads produced 4,234,386 contigs. Following gene prediction and redundancy elimination, a final non-redundant gene catalog comprising 7,287,124 genes was constructed.

### 3.4. Composition of Dominant Microbial Taxa

**Figure 3:**
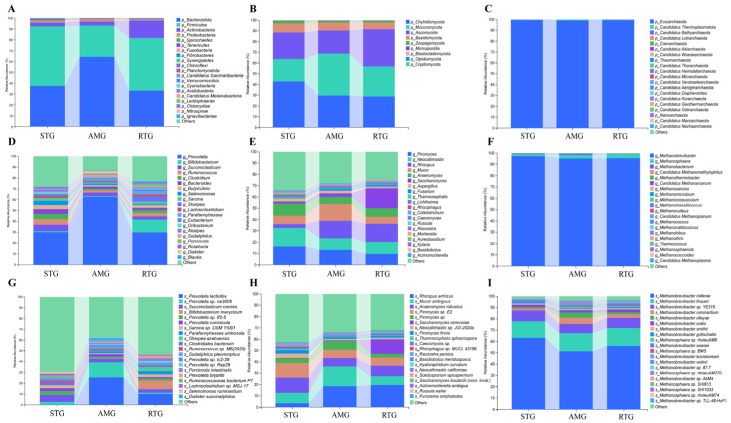
Top 20 dominant microbial genera at the phylum (**A**–**C**), genus (**D**–**F**) and species level (**G**–**I**); the relative abundance of microbiota is depicted on the ordinate, whereas the abscissa corresponds to the various experimental treatment groups ((**A**,**D**,**G**), bacteria; (**B**,**E**,**H**), fungi; (**C**,**F**,**I**), archaea). STG: lambs given a basal diet and 1 L saline in total; AMG: lambs given a basal diet with 15 g/d per lamb of *Allium mongolicum* Regel powder and 1 L saline in total; RTG: lambs given a basal diet and 1 L rumen fluid from donor sheep supplemented with 15 g/d of *Allium mongolicum* Regel.

At the phylum level, Firmicutes and Bacteroidota were the dominant bacteria across the STG, AMG, and RTG. Specifically, Firmicutes accounted for 56.98%, 48.55%, and 55.59% of the relative abundance in the STG, AMG, and RTG, whereas Bacteroidota comprised 32.05%, 36.96%, and 28.40%, respectively (Figure 3A). Regarding the fungal communities (Figure 3B), Chytridiomycota, Mucoromycota, Ascomycota, and Basidiomycota consistently maintained dominance across all groups. Their relative abundances in the STG were 42.88%, 20.97%, 24.65%, and 8.45%, respectively. These proportions shifted to 29.76%, 39.07%, 21.70%, and 7.36% in the AMG, and 28.85%, 27.96%, 34.72%, and 6.39% in the RTG. For archaea (Figure 3C), Euryarchaeota exhibited absolute dominance, representing 99.44%, 99.24%, and 99.48% of the communities in the STG, AMG, and RTG, respectively.

The dominant microbial genera at the genus level are shown in Figure 3D–F. *Prevotella*, *Bifidobacterium*, *Succiniclasticum*, *Ruminococcus*, and *Clostridium* were identified as the predominant bacterial genera across all groups. Their relative abundances were 29.92%, 9.83%, 5.98%, 4.94%, and 5.24% in the STG; 62.47%, 8.37%, 3.33%, 1.99%, and 1.60% in the AMG; and 29.75%, 12.09%, 2.75%, 3.01%, and 2.88% in the RTG, respectively (Figure 3D). For fungi, *Piromyces*, *Neocallimastix*, *Rhizopus*, *Mucor*, and *Anaeromyces* were dominant, with relative abundances of 16.27%, 16.42%, 3.31%, 7.39%, and 10.79% in STG; 13.31%, 10.28%, 15.56%, 14.77%, and 6.34% in AMG; and 9.71%, 10.52%, 16.15%, 6.12%, and 7.55% in RTG, respectively (Figure 3E). Regarding archaea, *Methanobrevibacter* and *Methanosphaera* were the two predominant genera, contributing 97.27% and 1.91% in STG, 95.19% and 3.41% in AMG, and 95.39% and 3.58% in RTG, respectively (Figure 3F).

At the species level (Figure 3G–I), the dominant bacterial taxa across the three groups were identified as *Prevotella lacticifex*, *Prevotella* sp. ne3005, *Succiniclasticum ruminis*, *Bifidobacterium merycicum*, and *Prevotella* sp. tf2-5 (Figure 3G). Their relative abundances in the STG were 0.14%, 2.72%, 6.29%, 0.42%, and 3.31%, respectively. In the AMG, the corresponding values were 25.48%, 13.77%, 3.65%, 0.42%, and 1.37%, while in the RTG, they accounted for 10.31%, 1.14%, 3.00%, 8.16%, and 1.89%. *Regarding fungi*, *Rhizopus arrhizus*, *Mucor ambiguus*, *Anaeromyces robustus*, *Piromyces* sp. E2, and *Piromyces* sp. were the predominant species (Figure 3H). Their proportions in the STG were 3.55%, 9.06%, 13.51%, 12.84%, and 4.36%, respectively; in the AMG, 18.32%, 17.63%, 7.66%, 6.94%, and 7.78%; and in the RTG, 19.71%, 7.51%, 9.48%, 7.03%, and 3.33%. Among archaea, the major taxa were *Methanobrevibacter millerae*, *Methanobrevibacter thaueri*, *Methanobrevibacter* sp. YE315, *Methanobrevibacter ruminantium*, and *Methanobrevibacter olleyae* (Figure 3I). Their respective abundances in the STG were 63.19%, 14.57%, 9.74%, 1.73%, and 1.07%. In the AMG group, they accounted for 51.16%, 16.13%, 8.28%, 5.67%, and 4.34%, while in the RTG, they represented 56.14%, 15.81%, 8.95%, 2.88%, and 2.15%.

### 3.5. Microbial Richness and Differential Taxonomic Abundance

**Figure 4:**
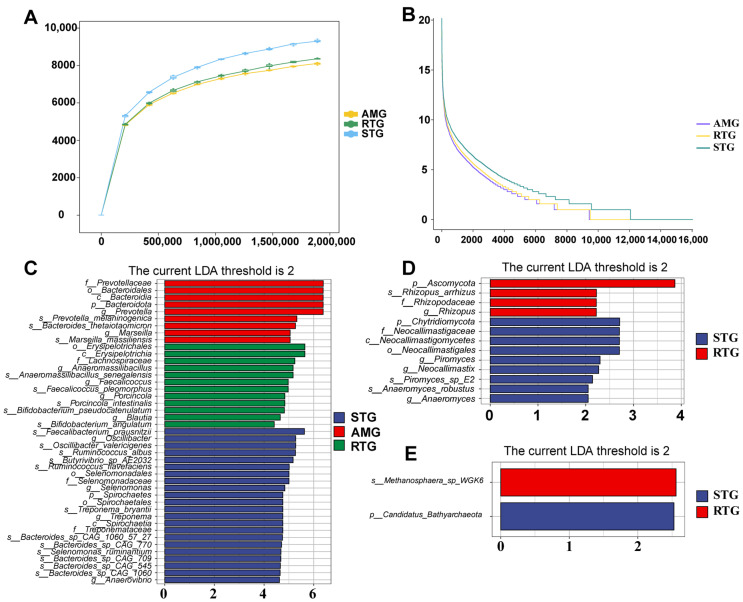
Microbial richness evaluation and identification of differentially abundant taxa. (**A**) Rarefaction curve: sequencing depth and species saturation; the x-axis shows the total number of sequences randomly sampled from each sample, and the y-axis shows the number of species observed at the corresponding sequencing depth. (**B**) Abundance-rank curve: species-level taxa abundance distribution; the x-axis shows species-level taxa ranked by abundance, and the y-axis displays the abundance of each species in a given sample. (**C**–**E**) LEfSe analysis revealed significant differences in bacterial taxa among the treatments ((**C**), bacteria; (**D**), fungi; (**E**), archaea); significantly different taxa are listed along the y-axis, with the x-axis displaying the logarithmic LDA scores for each identified taxon.

Microbial community structure in lambs varied markedly among treatments. Sequencing depth sufficiency and normalization were evaluated using rarefaction analysis: rarefaction curves of gene richness approached saturation for all treatment groups, indicating that sequencing depth was sufficient to cover most microbial diversity (Figure 4A), and observed species richness was consistently elevated in STG lambs compared to the AMG and RTG counterparts, with notable abundance disparities between STG and the treatment groups (Figure 4B).

To further identify robust taxonomic differences, LEfSe analysis was conducted on the abundance-filtered dataset (excluding taxa with relative abundance < 0.1%) to mitigate false positive discoveries. An overall Kruskal–Wallis test (*p* < 0.05) and subsequent Wilcoxon tests were applied among the three groups, followed by linear discriminant analysis (LDA score > 2.0). In total, 43 bacterial, 13 fungal, and 2 archaeal taxa were robustly discriminative among groups and are detailed in Figure 4C–E. For bacteria (Figure 4C), *Prevotella* and *Marseillia* showed higher abundance in the AMG; *Anaeromassilibacillus*, *Facalicoccus*, and *Porcincola* were more abundant in the RTG; and *Oscillibacter*, *Selenomonas*, and *Treponema* characterized the STG. In the fungal community (Figure 4D), representative taxa were detected mainly in the STG and RTG, with the RTG dominated by *Ascomycota* and *Rhizopus*, whereas the STG was characterized by *Piromyces* and *Neocallimastix*. For archaea (Figure 4E), *Methanosphaera* sp. WGK6 was enriched in the RTG, while *Candidatus Bathyarchaeota* was characteristic of the STG.

### 3.6. Correlation Analysis of Dominant Microbiota with Different Environment Factor

**Figure 5:**
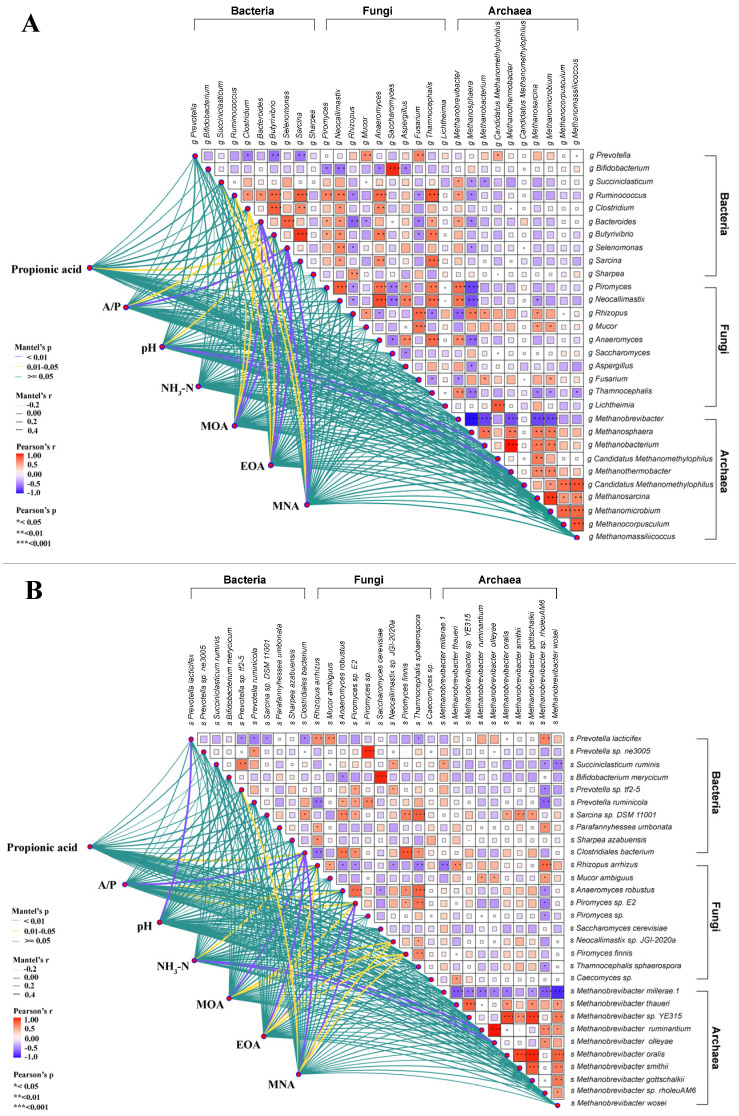
Assessment of the associations linking predominant bacterial communities with environmental variables. ((**A**), genus level; (**B**), species level). A/P: ratio of acetic acid to propionic acid; NH_3_-N: ammonia nitrogen; MOA: 4-methyloctanoic acid; EOA: 4-ethyloctanoic acid; MNA: 4-methylnonanoic acid. Asterisks *, **, and *** indicate *p* < 0.05, *p* < 0.01, and *p* < 0.001, respectively.

At the genus level (Figure 5A), bacterial genera generally exhibited positive associations with environmental factors, whereas fungal genera showed no clear correlations. Specifically, A/P showed a positive correlation with *Selenomonas* (R = 0.47, *p* = 0.005). pH showed a positive correlation with *Methanobacterium* (R = 0.48, *p* = 0.005) and with *Methanothermobacter* (R = 0.49, *p* = 0.008). MOA showed a positive correlation with *Bacteroides* (R = 0.43, *p* = 0.002) and with *Selenomonas* (R = 0.56, *p* = 0.002). EOA showed a positive correlation with *Bacteroides* (R = 0.40, *p* = 0.002) and with *Selenomonas* (R = 0.58, *p* = 0.001). MNA showed a positive correlation with *Bacteroides* (R = 0.42, *p* = 0.001) and with *Selenomonas* (R = 0.54, *p* = 0.002).

At the species level (Figure 5B), clear associations were identified between bacterial, fungal, and archaeal taxa and various environmental factors. Propionic acid showed a positive correlation with *Clostridiales bacterium* (R = 0.47, *p* = 0.010). A/P showed a positive correlation with *Clo. bacterium* (R = 0.53, *p* = 0.002). pH showed a positive correlation with *Pre. lacticifex* (R = 0.45, *p* = 0.007). NH_3_-N showed a positive correlation with *Met. ruminantium* (R = 0.47, *p* = 0.006) and with *Met. olleyae* (R = 0.48, *p* = 0.005). *Clo. bacterium* showed a positive correlation with MOA (R = 0.43, *p* = 0.004), EOA (R = 0.43, *p* = 0.003), and MNA (R = 0.42, *p* = 0.006).

### 3.7. Redundancy Analysis of Microbiota and Environmental Factors

**Figure 6:**
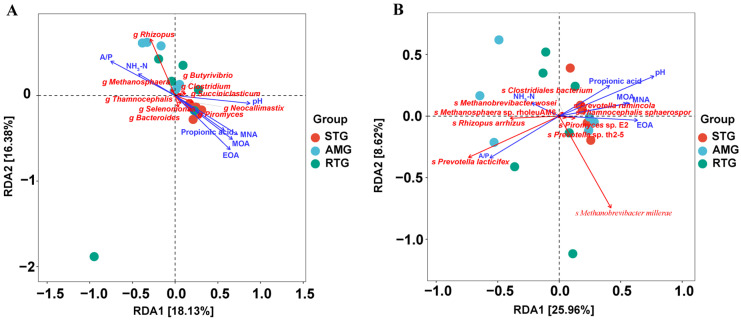
Redundancy analysis of the relationships between environmental factors and microbial communities. ((**A**), genus level; (**B**), species level). The horizontal and vertical axes represent the proportion of variation explained by RDA1 and RDA2, respectively. MOA: 4-methyloctanoic acid; EOA: 4-ethyloctanoic acid; MNA: 4-methylnonanoic acid. Colors indicate different experimental groups: red for STG (the lamb given a basal diet and administered 1 L saline in total; cyan for AMG) (the lamb given a basal diet with 15 g/d per lamb of *Allium mongolicum* Regel powder and administered 1 L saline in total); green for RTG (the lamb given a basal diet and administered 1 L rumen fluid from donor sheep supplemented with 15 g/d of *Allium mongolicum* Regel).

Redundancy analysis further examined the associations linking environmental factors to microbial communities (Figure 6). At the genus level (Figure 6A), the first two RDA axes explained 18.13% and 16.38% of the total variation, respectively. Among the environmental variables, pH (R^2^ = 0.52, *p* = 0.003), A/P (R^2^ = 0.47, *p* = 0.001), MNA (R^2^ = 0.47, *p* = 0.004), EOA (R^2^ = 0.45, *p* = 0.004) and MOA (R^2^ = 0.43, *p* = 0.006) had significant correlations with the community composition, whereas propionic acid (R^2^ = 0.30, *p* = 0.08) and NH_3_-N (R^2^ = 0.16, *p* = 0.27) did not. Samples from the STG were mainly ordinated towards higher levels of propionic acid, MOA, EOA, MNA and pH, and were characterized by the genera *Neocallimastix*, *Piromyces*, *Selenomonas*, and *Bacteroides*. In contrast, samples from the AMG and RTG were located in the opposite direction and were enriched in *Rhizopus*, *Butyrivibrio*, *Clostridium*, *Succiniclasticum* and *Methanosphaera*, which were positively associated with NH_3_-N and the A/P.

At the species level (Figure 6B), the first two axes explained 25.96% and 8.62% of the variation. Although these measured factors account for a modest proportion of the overall community variance, among the environmental variables, pH (R^2^ = 0.49, *p* = 0.01), whereas other environmental variables exhibited weaker and insignificant relationships (A/P: R^2^ = 0.30, *p* = 0.08; propionic acid: R^2^ = 0.16, *p* = 0.27; MOA: R^2^ = 0.22, *p* = 0.17). Sample ordination appeared more clustered for the STG, whereas AMG and RTG samples were more scattered along the RDA axes. Overall, these patterns suggest that, under the data resolution and sample size of the present study, relationships between microbial community composition and environmental factors are more clearly captured at the genus level. Species-level associations may be more dispersed or limited by insufficient statistical power, and therefore require further validation with larger sample sizes or higher sequencing depth.

### 3.8. Identification of Differential Metabolites

**Figure 7:**
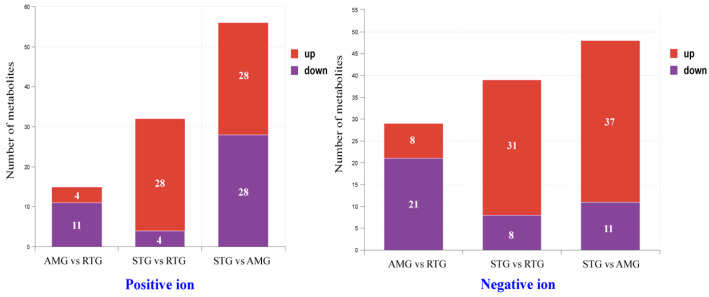
Screening of differential metabolites. Differential metabolites were filtered using thresholds of VIP > 1 and *p* < 0.05; the x-axis represents the comparison pairs, while the y-axis shows the number of identified metabolites. STG: the lamb given a basal diet and administered 1 L saline in total; AMG: the lamb given a basal diet with 15 g/d per lamb of *Allium mongolicum* Regel powder and administered 1 L saline in total; RTG: the lamb given a basal diet and administered 1 L rumen fluid from donor sheep supplemented with 15 g/d of *Allium mongolicum* Regel.

After FDR correction, no variables remained statistically significant (Figure 7; FDR > 0.1). Therefore, variables meeting the criteria of VIP > 1 combined with an uncorrected *p* < 0.05 were selected. It should be explicitly noted that these represent nominal findings and are utilized in this study strictly for exploratory interpretation of metabolic trends. Under the positive ion mode, 56 metabolites differed between the STG and RTG, with 28 metabolites upregulated in the STG and 28 downregulated in the RTG (Supplementary Table S1). A total of 32 metabolites differed between the STG and AMG, including 28 upregulated metabolites in the STG and 4 downregulated metabolites in the AMG (Supplementary Table S2). A total of 15 differential metabolites were identified between the AMG and RTG, with 4 upregulated in the AMG and 11 downregulated in the RTG (Supplementary Table S3).

Under the negative ion mode, 48 metabolites differed between the STG and AMG, with 37 upregulated and 11 downregulated in the AMG (Supplementary Table S4). Between the STG and RTG, 39 metabolites showed differences, including 31 upregulated metabolites in the STG and eight downregulated metabolites in the RTG (Supplementary Table S5). Between the AMG and RTG, 29 metabolites differed, with eight upregulated metabolites in the AMG and 21 downregulated metabolites in the RTG (Supplementary Table S6).

### 3.9. Differential Metabolites Analysis

**Figure 8:**
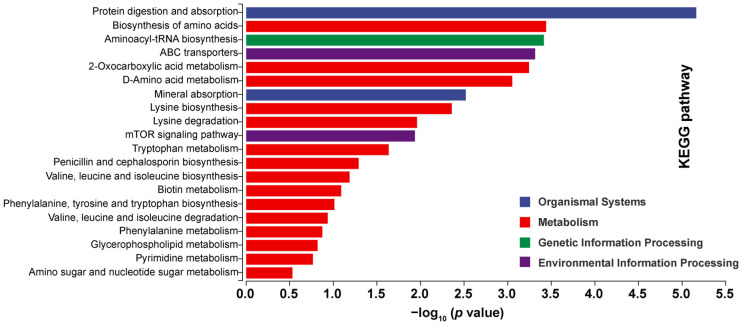
KEGG enrichment analysis of differentially expressed metabolites. The top 20 pathways with the *p* < 0.05 and FDR < 0.1, the most significantly enriched pathways, were selected for display: the x-axis shows −log_10_ (*p*-value), and the y-axis displays the KEGG pathways.

Under mixed ion mode, the KEGG pathways associated with the top 20 nominal differential metabolites (identified via VIP > 1 and uncorrected *p* < 0.05) were analyzed and are shown in Figure 8. KEGG functional classification revealed that there were significantly distinct metabolic routes across the three experimental groups (Supplementary Table S7). The differential metabolites were mainly annotated into Metabolism, succeeded by Organismal Systems, Environmental Information Processing, and Genetic Information Processing. Enrichment analysis further indicated that the STG, AMG, and RTG differed significantly in pathways related to Protein Digestion and Absorption (*p* < 0.001, FDR < 0.001), Biosynthesis of Amino Acids (*p* < 0.001, FDR < 0.001), Mineral Absorption (*p* < 0.001, FDR < 0.001), Aminoacyl-tRNA Biosynthesis (*p* < 0.001, FDR < 0.001), D-Amino Acid Metabolism (*p* < 0.001, FDR < 0.001), ABC Transporters (*p* < 0.001, FDR < 0.001), 2-Oxocarboxylic Acid Metabolism (*p* < 0.001, FDR < 0.001), Tryptophan Metabolism (*p* < 0.001, FDR < 0.001), Cysteine And Methionine Metabolism (*p* < 0.05, FDR < 0.001), and Lysine Degradation (*p* < 0.001, FDR = 0.003). These pathways are primarily involved in protein digestion and absorption, amino acid biosynthesis and catabolism, transmembrane transport, mineral uptake, energy metabolism, growth regulation via signaling, and specific amino acid pathways such as Lys and tryptophan metabolism.

### 3.10. Cluster Analysis of Differential Metabolites

**Figure 9:**
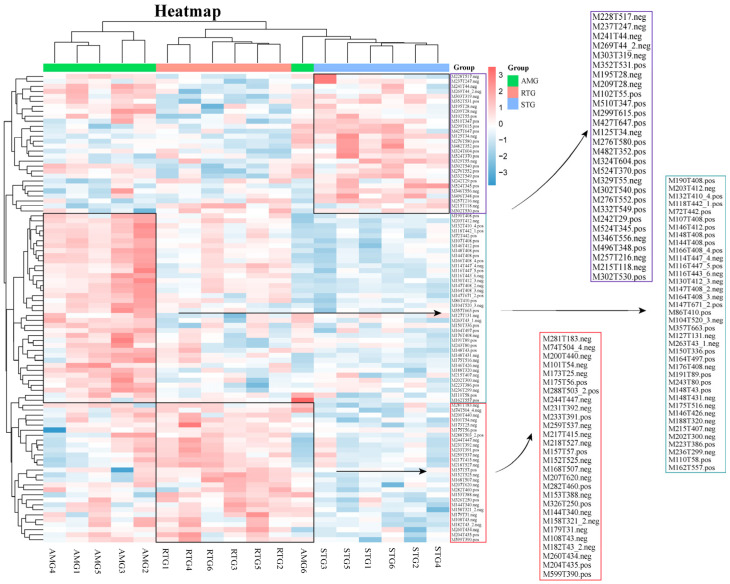
Hierarchical heatmap showing the clustering of differential metabolites. Red indicates higher and blue indicates lower expression levels. Columns represent individual samples, rows represent identified metabolites, and the dendrogram on the left shows clustering of differential metabolites. STG: the lamb given a basal diet and administered 1 L saline in total; AMG: the lamb given a basal diet with 15 g/d per lamb of *Allium mongolicum* Regel powder and administered 1 L saline in total; RTG: the lamb given a basal diet and administered 1 L rumen fluid from donor sheep supplemented with 15 g/d of *Allium mongolicum* Regel.

The cluster analysis results of hepatic metabolites are shown in Figure 9. The STG comprised 28 nominal metabolites, descriptively characterized by lipids and lipid-like molecules (28.57%), including M510T347 (1-heptadecanoyl-sn-glycero-3-phosphocholine), M482T352 (1-hexadecyl-sn-glycero-3-phosphocholine), M524T345 (1-Stearoyl-sn-glycerol 3-phosphocholine), M496T348 (1-palmitoyl-sn-glycero-3-phosphocholine), M257T216 (Tetradecanedioic acid), M329T55 (11beta-hydroxyprogesterone), and M276T552 (L-glutarylcarnitine). The AMG comprised 38 putative characteristic metabolites, primarily characterized by organic acids and their derivatives (39.47%), including M190T408 (Gly-Phe-Arg), M132T410_4 (Leucine), M144T408 (Met-His), M166T408_4 (Phe), M114T447_4 (DL-proline), M116T447_5 (DL-arginine), M130T412_3 (Norleucine), M164T408_3 (DL-phenylalanine), M147T671_2 (Lys), M104T520_3 (DL-serine), M176T408 (2-Oxoadipic acid), M191T89 (Thr-Ala), M148T431 (L-methionine), M146T426 (N-acetyl-dl-serine), and M188T320 (Asn-Gly). Similarly, the RTG comprised 28 such metabolites, primarily characterized by organic acids and their derivatives (53.57%), including M74T504_4 (Glycine), M200T440 (Cysteine-s-sulfate), M173T25 (Isocitric acid), M175T56 (Val-Gly), M244T447 (Ile-Asn), M231T392 (Ile-Thr), M217T415 (Val-Thr), M218T527 (Ser-Asn), M152T525 (L-Cysteinesulfinic acid), M168T507 (L-Cysteic acid), M207T620 (Dl-lanthionine), M144T340 (Isobutyrylglycine), M158T321_2 (Isovalerylglycine), M260T434 (O-phosphotyrosine), and M599T390 (Phe-met-arg-phe-amide).

### 3.11. Correlation Between Key Microbiota and Metabolites Affecting KBCFA Deposition

**Figure 10:**
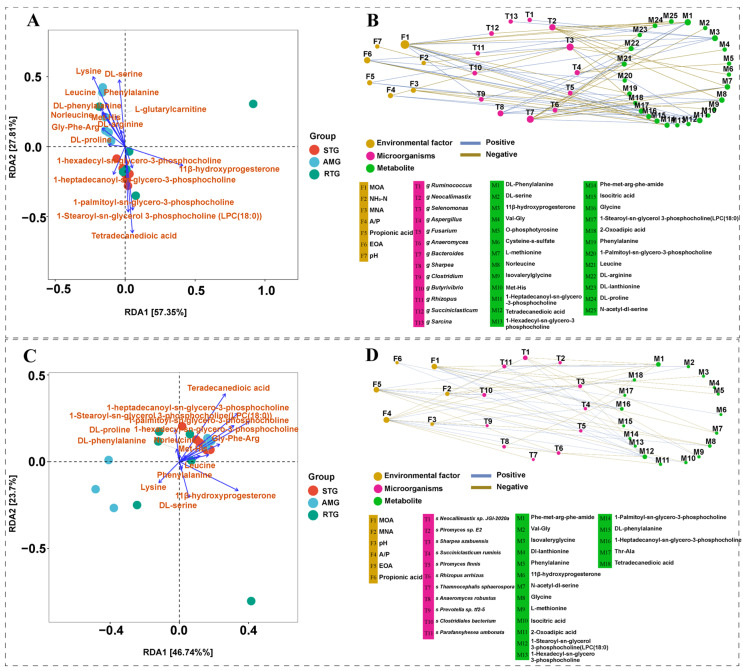
Correlation network of key microbes and branched-chain fatty acid-related metabolites and redundancy analysis. ((**A**,**B**), genus level; (**C**,**D**), species level). In RDA analysis, the horizontal and vertical axes represent the percentage of variation accounted for by RDA1 and RDA2, respectively. In networks, blue lines represent positive correlations, while yellow lines denote negative ones. Node size reflects association number. Spearman correlation analysis (|R| ≥ 0.6, *p* < 0.05). MOA refers to 4-methyloctanoic acid; EOA refers to 4-ethyloctanoic acid; MNA refers to 4-methylnonanoic acid. Different colors denote different groups: red for STG (the lamb given a basal diet and administered 1 L saline); cyan for AMG (the lamb given a basal diet with 15 g/d per lamb of *Allium mongolicum* Regel powder and administered 1 L saline); green for RTG (the lamb fed with a basal diet and received total of 1 L rumen fluid from donor sheep supplemented with 15 g/d of *Allium mongolicum* Regel).

To explore the potential statistical associations between rumen microbiota and hepatic metabolites, we employed both multivariate RDA and pairwise spearman correlation analysis (|R| ≥ 0.6, *p* < 0.05). RDA was conducted to visualize the overall relationships between the microbial community structure and metabolic profiles. At the genus level (Figure 10A), the selected metabolites explained 85.16% of the total variance, indicating a strong statistical correlation between specific metabolic factors and microbial distribution. The RDA biplot revealed distinct clustering patterns: the AMG was closely associated with amino acid-related metabolites, such as Lysine, Leucine, and Phenylalanine, whereas the STG clustered predominantly lipid-related metabolites, such as 1-Stearoyl-sn-glycerol-3-phosphocholine (LPC(18:0)) and Tetradecanedioic acid. A similar pattern was observed at the species level (Figure 10C), further supporting the co-occurrence of specific taxa and metabolic phenotypes. Subsequently, Spearman correlation analysis was performed on the top 20 dominant taxa (|R| ≥ 0.6, *p* < 0.05). The results indicated significant statistical associations. At the genus level (Figure 10B), MOA was negatively correlated with *Clostridium*, *Butyrivibrio*, and *Succiniclasticum*, as well as with metabolites such as DL-Phenylalanine, Phe-met-arg-phe-amide, and Phe. Conversely, it exhibited positive correlations with lipid metabolites like 1-Stearoyl-sn-glycerol-3-phosphocholine (LPC(18:0)). MNA showed negative correlations with Isovalerylglycine and Glycine. EOA was positively correlated with *Selenomonas*, Met-His, and Glycine, but negatively with Isocitric acid. At the species level (Figure 10D), consistent patterns were observed. MOA displayed negative associations with Phe-met-arg-phe-amide and Phe, while correlating positively with LPC(18:0). MNA exhibited positive correlations with Isovalerylglycine and *Neocallimastix* sp. JGI-2020a. EOA was inversely correlated with *Sharpea azabuensis* and *R. arrhizus*, while positively correlated to *Pir.* sp. E2 and LPC(18:0). However, the observed associations suggest a complex metabolic interplay or shared responses to environmental factors, which warrants further functional verification.

## 4. Discussion

### 4.1. Rumen Fermentation

In this study, the A/P values for AMG and RTG surpassed those of the STG significantly. This phenomenon not only suggests a potential improvement in the digestibility of structural carbohydrates and a relative reduction in the utilization of non-structural carbohydrates [25], but also aligns well with the hepatic metabolite profile and the observed associations between the microbiome and metabolome. The characteristic metabolites in the STG predominantly consisted of lipids and lipid-like compounds, while the AMG and RTG were markedly enriched in organic acids and their derivatives, especially various amino acids and dipeptides. This shift in hepatic metabolism from being lipid-dominated to amino-acid or organic-acid-dominated statistically parallels the increased A/P and the apparent shifts in protein or nitrogen metabolism. Together, these correlations lead to the hypothesis that the active components in AMR may be associated with the distribution of energy and nitrogen metabolism in the body, potentially linked to altered rumen fermentation products.

Specifically, although NH_3_-N levels in the AMG and RTG were increased, they remained within the physiological range [26]. Together with the enrichment of various amino acids and sulfur-containing amino acid derivatives in the liver, this leads to the hypothesis that, under AMR supplementation and RFT, ruminal NH_3_-N might be efficiently utilized by microbiota for microbial protein synthesis. Although microbial protein flow was not directly measured in the current study, such a dynamic would be consistent with the observed enhanced accumulation and transport of amino acids and their derivatives at the hepatic level. In contrast, although the STG showed lower NH_3_-N concentrations, its characteristic hepatic metabolites were mainly phospholipids and fatty acid derivatives, suggesting a metabolic pattern in which microbial protein synthesis may be limited and the animal relies more on lipid mobilization and oxidation to sustain growth [27,28].

The correlation analysis further provided contextual support for the potential links along the microbiota–metabolite–KBCFA axis. MOA was negatively correlated with typical butyrate- and propionate-producing bacteria such as *Clostridium* and *Butyrivibrio*, as well as with amino acids including DL-phenylalanine and Phe, but positively correlated with lipid molecules such as 1-heptadecanoyl-sn-glycero-3-phosphocholine and 1-hexadecyl-sn-glycero-3-phosphocholine. This statistical association suggests a potential pattern where high MOA levels coincide with enhanced lipid-related metabolism and relatively insufficient amino acid utilization, resembling the lipid-dominated metabolic pattern observed in the STG. In contrast, in the AMG and RTG, MOA was downregulated and showed negative correlations with LPC-type lipids but was aligned with the changes in amino acids and dipeptides, which is more consistent with an amino acid- and organic acid-dominated metabolic profile. Meanwhile, MNA was negatively correlated with intermediates related to branched-chain amino acid or nitrogen metabolism, such as isovalerylglycine and glycine. This further suggests the hypothesis that supplementation with AMR and RFT may enhance the efficiency of nitrogen conversion into a body-available amino acid pool by modulating the balance between the production and breakdown of amino acids by rumen microbes. Meissner et al. [29] have shown that excessive accumulation of propionate is often accompanied by subclinical ruminal acidosis and reduced pH, and that bacteria involved in propionate production genera such as *Prevotella* and *Fibrobacter* have a significant role in this process [30]. In this study, AMR supplementation and RFT, on the one hand, reduced the abundance and functional potential of bacteria closely associated with propionate production, which coincided with a moderate decrease in the propionate proportion. On the other hand, EOA was positively correlated with *Selenomonas* and amino acids such as glycine and Phe, but negatively correlated with isocitric acid. This implies a possible mechanism where, under an acetate-dominated fermentation pattern, tricarboxylic acid (TCA) cycle intermediates such as isocitric acid might be diverted to support amino acid synthesis and nitrogen metabolism rather than simply entering complete oxidative pathways. Ding et al. [31] reported that phenolic and flavonoid compounds in AMR may selectively suppress certain propionate- or lactate-producing bacteria and lipid-mobilizing taxa, while enhancing the combined activity of fiber-degrading bacteria and those involved in protein and amino acid metabolism. This is hypothesized to shift rumen fermentation from a pattern of high propionate and low acetate toward a more balanced state with moderately elevated acetate and controlled propionate levels, thereby potentially helping to maintain a more stable rumen pH and fermentation environment. Building on this hypothesis, an adequate NH_3_-N supply, together with putatively more efficient microbial protein synthesis, enables a larger proportion of nitrogen to reach the liver as amino acids and dipeptides. This literature-based theoretical framework strongly aligns with our current observations in the hepatic metabolome, where we found an increased proportion of organic acids and their derivatives, particularly amino acids, sulfur-containing amino acids and their conjugates, among the AMG and RTG, accompanied by a relative reduction in lipids and lipid-like molecules.

### 4.2. Factors in the Biosynthesis of Gamey Flavor

In sheep meat, KBCFA are key contributors to the characteristic gamey flavor [32,33]. These compounds are directly linked to consumer rejection of mutton products. Their sensory thresholds vary significantly: MNA at 0.65 ppm, MOA at 0.02 ppm, and EOA at 0.006 ppm [34], indicating that EOA is the most potent contributor to off-flavors. Previous studies have demonstrated the inhibitory effect of AMR extracts on mutton flavor; however, the synergistic effects of the complex bioactive compounds in AMR remained to be elucidated. This study confirms that both AMR feeding and RFT were shown to significantly reduce KBCFA concentrations in lamb meat. We propose that this reduction is potentially linked to the modulation of specific rumen microbial pathways by AMR bioactives [8]. Crucially, the synthesis of MOA and MNA is closely linked to propionate metabolism: acetyl-CoA undergoes chain elongation via methylmalonyl-CoA, a pathway that utilizes propionate as a key substrate [35,36]. AMR supplementation and RFT significantly reduced the levels of propionate in the rumen. This reduction in precursor availability likely contributes to the suppression of the downstream synthesis of MOA and MNA. Additionally, rumen microbes utilize branched-chain amino acids as precursors for KBCFA synthesis [37]. The observed potential shift in metabolic flux, where amino acids are preserved and transported to the liver rather than degraded into volatile fatty acids, could further limit the substrate pool for KBCFA formation. Therefore, AMR appears to alter rumen metabolism to favor protein synthesis and acetate production over propionate. This metabolic shift is likely responsible for reducing the available substrates needed to make gamey flavor compounds.

### 4.3. Modulation of Ruminal Microbiota by AMR Supplementation and RFT

The digestive and metabolic pathways in the rumen are highly complex, relying on specialized microbial consortia that drive host nutrient absorption and phenotypic traits [38,39]. Metagenomic analysis revealed that AMR feeding significantly altered the phylum-level abundance of dominant bacterial taxa, with the four predominant groups being *Bacteroidota*, *Firmicutes*, *Actinobacteria*, and *Proteobacteria*. Specifically, AMR supplementation increased the relative abundance of *Bacteroidota* by 42.19% while decreasing *Firmicutes* by 47.27%. While *Bacteroidota*-dominated communities are conventionally associated with propionate production [40], the metabolic phenotype observed in this study indicates a functional redirection. *Bacteroidota*, particularly the genus *Prevotella*, possesses a broad enzymatic spectrum for degrading hemicellulose and, crucially, proteins [41]. Therefore, the expansion of *Bacteroidota* in the AMG and RTG likely contributed to enhanced proteolytic activity and nitrogen metabolism efficiency. This aligns perfectly with the enrichment of amino acids and dipeptides observed in the hepatic metabolome, rather than being strictly associated with propionate accumulation. Regarding diversity, a decline in species richness and evenness was evident in both AMG and RTG. This effect is hypothesized to be attributable to the antimicrobial properties of polyphenolic compounds and terpenoids in AMR. Similar to garlic powder supplementation and other plant flavonoids [42,43], bioactives in AMR may selectively suppress specific microbial populations in a concentration-dependent manner, thereby reshaping the community structure.

LEfSe analysis provided key information on the potential mechanisms underlying propionate reduction. Crucially, the STG was enriched with *Selenomonadaceae*, a taxon well-known for converting succinate to propionate via the methylmalonyl-CoA pathway [44]. The greater abundance of *Selenomonadaceae* in the STG may partially explain the lower A/P ratio and the subsequent accumulation of propionate-derived MOA and MNA. Additionally, the STG was enriched with fiber-degrading fungi such as *Piromyces* and *Neocallimastix*. While these fungi secrete enzymes for plant cell wall degradation [45], their co-occurrence with propionate producers suggests a fermentation pattern in the STG that directs carbon flux toward lipid mobilization rather than protein deposition. In contrast, the AMG was dominated by *Prevotella* and *Bacteroides*. *Prevotella* is proficient in protein and hemicellulose degradation. Its dominance, coupled with the suppression of the propionate producing *Selenomonadaceae*, supports a fermentation shift where reduced propionate production limits the substrate availability for KBCFA synthesis, while enhanced proteolysis supports the host’s amino acid pool. In the RTG, genera such as *Anaeromassilibacillus* and *Faecalicoccus* were dominant, potentially contributing to short-chain fatty acid balance and energy supply. Correlation analysis further hinted at inter-domain interactions. For instance, *Selenomonas* was positively correlated with *Neocallimastix* in the STG context, suggesting a potential synergistic relationship that supports the propionate heavy fermentation profile.

### 4.4. Differential Metabolites and Metabolic Pathways

Correlation and cluster analyses revealed that AMR supplementation and RFT were associated with a shift in the hepatic metabolome from a lipid-dominated profile to one centered on amino acid and energy metabolism. While KBCFA primarily originate from rumen microbial fermentation, hepatic metabolism has a critical gatekeeping role by determining whether BCAAs and absorbed lipids are deposited in tissue or oxidized for energy [46,47]. In the present study, the AMG and RTG were characterized by elevated organic acids and amino acid derivatives, coinciding with reduced KBCFA deposition in muscle. This matches a metabolic shift where more carbon and nitrogen are used to build protein instead of making fat, BCAAs are pivotal in this divergence. In the liver, BCAAs can either be incorporated into proteins or catabolized by branched-chain α-keto acid dehydrogenase into acyl-CoA precursors for fatty acid elongation [48]. The observed enrichment of amino acids and dipeptides in the AMG or RTG suggests that nitrogen is conserved in the amino acid pool. Specifically, AMR increased the hepatic abundance of Phe and Lys. Phe derivatives are known to activate the mTORC1 signaling pathway, which promotes protein synthesis while simultaneously suppressing lipogenesis [49]. Furthermore, elevated Phe may induce negative feedback on the complex responsible for branched-chain α-keto acid dehydrogenation via dopamine analog production, thereby limiting transforming BCAAs into their corresponding branched-chain acyl-CoA derivatives primers required for KBCFA elongation [50]. Additionally, the increase in L-Met suggests a potential for enhanced generation of S-adenosylmethionine. Based on existing literature [51,52], we hypothesize that this methyl donor might epigenetically modulate lipid regulators like PPARγ, which could theoretically suppress adipogenic gene expression and lipid extension; however, these gene targets were not directly measured in this study. In contrast, the STG hepatic metabolome was dominated by lipids such as 1-heptadecanoyl-sn-glycero-3-phosphocholine, facilitating lipid storage. MOA was positively correlated with LPCs and steroid hormones, markers of active lipogenesis. AMR supplementation appeared to disrupt this pattern, which we speculate might involve the suppression of SREBP-1c transcriptional activity and downstream fatty acid synthase expression as described in other models [53]. Conversely, the RTG showed elevated L-glutarylcarnitine, a metabolite that activates CPT-1 to enhance mitochondrial β-oxidation [54]. While CPT-1 activity was not assessed here, this profile leads to the hypothesis that RFT might help shift the liver to catabolize fatty acids rather than store them. The negative correlation between transplantation and acyl-CoA synthetase further aligns with a potential reduction in lipid accumulation efficiency. These results raise the possibility that AMR and RFT reduce gamey flavor not only by lowering the rumen production of KBCFA, but also by promoting pathways associated with fat burning in the liver and shifting metabolism toward making more protein.

Energy metabolism pathways, including the TCA cycle, also appear to play a regulatory role. Theoretically, enhanced TCA cycle activity could compete for Acyl-CoA substrates, potentially limiting their availability for fatty acid elongation [55]. The observed inverse relationship between EOA and isocitric acid implies that in the AMR group or RTG, precursors like isovaleryl-CoA might be preferentially channeled into the TCA cycle [56]. This hypothesized oxidative shift could be supported by the antioxidant properties of AMR bioactives, which have been reported to protect key TCA enzymes like succinate dehydrogenase [57]. Furthermore, in the RTG, the enrichment of rumen-derived glycine and the presence of butyrate producing *Butyrivibrio* present an interesting avenue for future research, as these factors could potentially modulate mitochondrial function through epigenetic mechanisms such as histone deacetylase inhibition, thereby theoretically optimizing the metabolic partitioning of carbon away from KBCFA synthesis.

**Figure 11:**
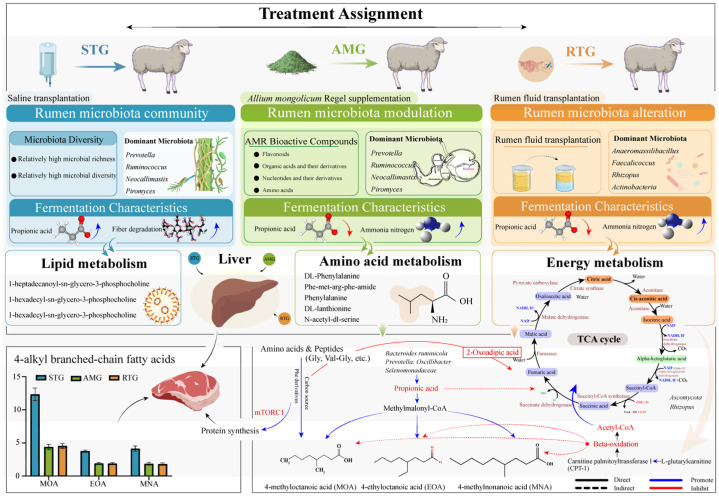
Elucidation of the core microbiota involved in 4-alkyl branched-chain fatty acid synthesis and the potential metabolic network pathways in the liver. Solid lines show direct interactions, while dashed lines indicate indirect ones. Blue denotes promoting processes or effects, whereas red represents inhibitory processes or effects.

Despite these biologically plausible linkages along the rumen–liver–muscle axis, it is important to acknowledge that the multi-omics correlation networks constructed in this study are fundamentally associative. While they provide robust hypotheses regarding how AMR and RFT modulate carbon and nitrogen fluxes to reduce KBCFA synthesis, they do not inherently establish biological directionality or direct causality. Future in vivo isotopic tracing or targeted in vitro functional validations are warranted to definitively confirm these microbiota–metabolite causal interactions. In summary, the reduction in gamey flavor is a systemic outcome: AMR modulates the rumen microbiota to reduce propionate and KBCFA production, while simultaneously reprogramming the hepatic metabolism to favor protein synthesis and fatty acid oxidation over lipogenesis. The proposed mechanism illustrating these dynamic changes across the gut–liver axis is summarized in Figure 11.

### 4.5. Limitation

Finally, it is important to acknowledge the inherent limitations of the experimental design, particularly regarding the RFT model. While RFT was utilized to investigate microbiota-mediated effects, rumen fluid is a complex matrix. It contains not only the microbial community but also microbial-derived metabolites, volatile fatty acids, and potentially residual bioactive compounds from the AMR diet. Because no specific control was included to account for this non-microbial carryover, the observed effects in the RTG cannot be exclusively attributed to the microbes alone. Therefore, the reduction in KBCFA should be cautiously interpreted as a partial microbiota-mediated contribution, working in tandem with the transferred metabolic milieu. Future studies utilizing germ-free models or isolated microbial consortia are required to definitively untangle the direct microbial effects from the metabolite carryover. Additionally, while our sub-cohort for metagenomics and metabolomics (*n* = 6 per group) was strictly selected based on PCoA centroids to maximize group representation, the relatively small sample size inherently limits overall statistical power. Consequently, the multi-omics correlations, LEfSe analyses, and proposed metabolic pathways presented in this study should be interpreted as exploratory rather than definitively confirmatory, providing a valuable framework for future studies with larger cohorts to validate these findings.

## 5. Conclusions

Supplementation with 15 g/d per lamb of *Allium mongolicum* Regel and *Allium mongolicum* Regel-based rumen fluid transplantation effectively reduced gamey 4-alkyl branched-chain fatty acids in lamb muscle. This reduction was associated with specific alterations within the microbiota-hepatic axis: *Allium mongolicum* Regel enriched amino acid-metabolizing taxa and derivatives, while transplantation promoted energy-metabolizing bacteria and metabolites commonly associated with lipid catabolism. Together, these observed metabolic profiles suggest a potential rerouting of precursors away from lipid synthesis, which may contribute to limiting off-flavor deposition. This highlights a novel dietary-microbial strategy to potentially reduce gamey flavor.

## Figures and Tables

**Table 1:** Ingredient formulation and chemical composition of the basal ration (on a dry matter basis).

Items (Basal Diet)	Content	Items (*Allium mongolicum* Regel)	Content
Ingredients		Chemical composition ^3^	
Corn stalk silage	19.67	Flavonoids	24.33
Alfalfa meal	16.35	Organic acids and their derivatives	20.02
Corn stalk	10.01	Nucleotides and their derivatives	12.87
Wheat bran	7.30	Amino acids	14.07
Corn	25.20	Hydroxycinnamoyl derivatives	5.22
Soybean meal	14.53	Amino acids derivatives	3.41
Extruded soybeans	1.83	Phenol amine	2.37
Premixes ^1^	2.48	Vitamins	1.40
NaCl	1.50	Choline	1.09
Limestone	1.13	Lipids	3.96
Total	100.00	Quinic acids and their derivatives	0.49
Nutrient levels		Others	10.77
Dry matter	86.13		
Metabolizable Energy ^2^ (MJ, kg)	11.82		
Crude protein	14.50		
Ether extract	2.50		
NDF	34.60		
ADF	16.43		
OM	85.00		
Ca	2.00		
P	0.80		

^1^ The premix supplied the following mineral elements per kg of the complete diet: 25.00 mg Fe (ferrous sulfate), 29.00 mg Zn (zinc sulfate), 30.00 mg Mn (manganese source), 8.00 mg Cu (copper sulfate), 0.45 mg I (potassium iodide), and 0.10 mg Co (cobalt sulfate). Dietary vitamin supplementation included 1200 IU of vitamin D, 20 IU of vitamin E, and 3200 IU of vitamin A. ^2^ Values for metabolizable energy were calculated, while the concentrations of all other nutrients were derived from analytical data. ^3^ The chemical profile of AMR was ascertained via laboratory analysis.

**Table 2:** Effects of *Allium mongolicum* Regel supplementation or rumen fluid transplantation on 4-alkyl branched-chain fatty acid levels in the *longissimus thoracis* muscle of lambs (mg/kg) ^1^.

Items	Treatments ^2^			SEM	*p*-Value
	STG	AMG	RTG		
MOA	12.34 ^a^	4.38 ^b^	4.55 ^b^	0.71	<0.001
EOA	4.13 ^a^	1.87 ^b^	1.81 ^b^	0.20	<0.001
MNA	3.77 ^a^	1.91 ^b^	1.90 ^b^	0.16	<0.001

^1^ The phenotypic KBCFA concentrations presented in this table were derived from the same animal trial and initially reported in our parallel study investigating meat quality [24]. They are included here to provide the biological context for the subsequent mechanistic analyses. ^2^ STG: given a basal diet and 1 L saline in total; AMG: given a basal diet with 15 g/d per lamb of *Allium mongolicum* Regel powder and 1 L saline in total; RTG: given a basal diet and 1 L rumen fluid from donor sheep in total; MOA: 4-methyloctanoic acid; EOA: 4-ethyloctanoic acid; MNA: 4-methylnonanoic acid. Values in the same row with different superscript letters indicate statistically significant differences (*p* < 0.05).

## Data Availability

The original contributions presented in this study are included in the article/Supplementary Materials. Further inquiries can be directed to the corresponding author.

## Supplementary Materials

The following supporting information can be downloaded at: https://www.mdpi.com/article/10.3390/foods15101617/s1, Table S1: Analysis of differential metabolites between the STG and RTG groups in positive ion mode. Table S2: Analysis of differential metabolites between the STG and AMG groups in positive ion mode. Table S3: Analysis of differential metabolites between the AMG and RTG groups in positive ion mode. Table S4: Analysis of differential metabolites between the STG and AMG groups in negative ion mode. Table S5: Analysis of differential metabolites between the STG and RTG groups in negative ion mode. Table S6: Analysis of differential metabolites between the AMG and AMG groups in negative ion mode. Table S7: KEGG enrichment analysis of the top 20 nominal differential metabolites.

## Abbreviations

The following abbreviations are used in this manuscript:

ADF	Acid Detergent Fiber
AMR	*Allium mongolicum* Regel
Ca	Calcium
CP	Crude Protein
CPM	Counts Per Million
DM	Dry Matter
EE	Ether Extract
EI	Electron Ionization
EOA	4-ethyloctanoic Acid
FID	Flame Ionization Detector
GC-MS	Gas Chromatography-mass Spectrometry
KBCFA	Three Key 4-alkyl Branched-chain Fatty Acids
KO	KEGG Orthology
LOQ	Limits of Quantification
LT	*Longissimus Thoracis*
MNA	4-methylnonanoic Acid
MOA	4-methyloctanoic Acid
NDF	Neutral Detergent Fiber
NH3-N	Ammonia Nitrogen
ORFs	Open Reading Frames
P	Phosphorus
RFT	Rumen Fluid Transplantation
RSD	Relative Standard Deviations
SRA	Sequence Read Archive
TCA	Tricarboxylic Acid
VFAs	Volatile Fatty Acids
