# Supplementary material for: Allium mongolicum Regel-Mediated Rumen Microbiota Intervention Modulates Hepatic Metabolome to Reduce 4-Alkyl Branched-Chain Fatty Acids in Lamb Longissimus Thoracis Muscle
Source: Foods. 2026 May 7;15(10):1617. doi: 10.3390/foods15101617 (PMC13206602; doi:10.3390/foods15101617)
Supplement: Supplementary file 1 [file foods-15-01617-s001.zip › Supplementary Table S1.pdf]

Supplementary Table S1: Analysis of differential metabolites between the STG and RTG groups in positive ion mode.

ID	Name	Mean-STG	Mean-RTG	FC	log_2FC	*P*-value	VIP	Regulation
M102T444	Betaine aldehyde	37484486.48	54925220.40	1.47	0.55	0.0473	1.73	Up
M107T408	Methyl 4,6-o-benzylidene-.alpha.-d-glucopyranoside	40880491.25	45400837.49	1.11	0.15	0.0335	1.90	Up
M116T447_5	DL-arginine	7179474987.68	7897171754.98	1.10	0.14	0.0329	1.87	Up
M124T346	4-Pyridinecarboxylic acid	14811963.34	21100898.34	1.42	0.51	0.0286	1.88	Up
M125T518_2	Cadaverine	13646364.08	19165511.06	1.40	0.49	0.0353	1.86	Up
M132T410_4	Leucine	10543719849.64	11446514801.44	1.09	0.12	0.0092	2.13	Up
M144T408	Met-His	182532962.95	200272168.60	1.10	0.13	0.0382	1.86	Up
M146T412	Oxyquinoline	53568512.57	61230506.48	1.14	0.19	0.0253	2.06	Up
M148T408	4,4'-bis(dimethylamino)benzophenone	10018412.89	11176639.23	1.12	0.16	0.0270	1.95	Up
M157T57	Indole-3-acetonitrile	5545332.53	8701808.74	1.57	0.65	0.0034	2.32	Up
M162T557	DL-2-Aminoadipic acid	97045105.18	112880510.90	1.16	0.22	0.0140	2.09	Up
M166T408_4	Phenylalanine	6125036408.68	6806020312.15	1.11	0.15	0.0118	2.11	Up
M175T441	Gly-Val	53024811.64	93698737.46	1.77	0.82	0.0030	2.34	Up
M175T56	Val-Gly	57412923.80	115357057.98	2.01	1.01	0.0037	2.29	Up
M189T441_2	Gly-Leu	71432195.44	96465001.86	1.35	0.43	0.0112	2.11	Up
M190T408	Gly-Phe-Arg	14705790.18	16339648.42	1.11	0.15	0.0205	1.97	Up
M191T516	L-hydroxyarginine	4218642.39	5730218.21	1.36	0.44	0.0449	1.77	Up
M204T435	N,n'-diacetylchitobiose	7695995.40	11171440.48	1.45	0.54	0.0366	1.86	Up
M233T391	Leu-Thr	49321008.34	89750405.41	1.82	0.86	0.0043	2.30	Up
M245T336	Leu-Ile	61890017.08	101006492.68	1.63	0.71	0.0298	1.89	Up
M282T460	1-methyladenosine	32731716.25	41938633.61	1.28	0.36	0.0349	1.96	Up
M288T503_2	Arg-Leu	8388254.92	14940636.69	1.78	0.83	0.0015	2.46	Up
M290T546_3	Adipoyl-l-carnitine	17229259.06	21436277.08	1.24	0.32	0.0433	1.78	Up
M293T445	Karanjin	16063445.26	21926372.16	1.36	0.45	0.0326	1.85	Up
M326T250	3'-O-methyladenosine	10081976.63	24372462.81	2.42	1.27	0.0104	2.12	Up
M448T390	Glycocholate	11681817.53	83441164.96	7.14	2.84	0.0462	1.75	Up
M599T390	Phe-met-arg-phe-amide	1508576.61	5497977.87	3.64	1.87	0.0425	1.76	Up
M86T410	Piperidine	1167747387.43	1314945390.40	1.13	0.17	0.0083	2.16	Up
M100T131	2-pyrrolidinone, 1-methyl-	190815751.92	113139110.23	0.59	-0.75	0.0401	1.83	Down
M162T519	L-Carnitine	135724102.03	99463214.20	0.73	-0.45	0.0377	1.79	Down
M171T518_1	alpha-N-Acetyl-L-glutamine	185187236.25	165700977.78	0.89	-0.16	0.0273	1.93	Down
M244T44	Myristoleic acid	21354499.57	13776124.33	0.65	-0.63	0.0151	2.02	Down
M247T43	Oleic acid	21990327.19	18533646.06	0.84	-0.25	0.0424	1.87	Down
M260T382	Hexanoyl-l-carnitine	95896140.93	48057810.21	0.50	-1.00	0.0277	1.89	Down
M261T496	Fenspiride	18600145.82	11696699.30	0.63	-0.67	0.0323	1.85	Down
M276T552	L-glutarylcarnitine	156845340.39	114928636.16	0.73	-0.45	0.0326	1.87	Down
M276T580	Glu-gln	135133513.46	70127023.82	0.52	-0.95	0.0119	2.20	Down
M277T615	Gamma-l-glutamyl-l-glutamic acid	200048846.09	140577935.46	0.70	-0.51	0.0217	2.03	Down
M290T546_2	Norcocaine	32596674.48	15949462.80	0.49	-1.03	0.0396	1.79	Down
M293T42	.delta.2-cis-eicosenoic acid	7086085.84	5457239.66	0.77	-0.38	0.0322	1.86	Down
M299T615	Mefenacet	9177915.37	7266458.78	0.79	-0.34	0.0139	2.11	Down
M302T540	Tyr-Arg	12381489.23	4229947.02	0.34	-1.55	0.0116	2.09	Down
M324T604	Cytidine 5'-monophosphate	9351397.61	5039944.57	0.54	-0.89	0.0234	1.96	Down
M332T549	Alitame	50037109.17	20245609.67	0.40	-1.31	0.0193	1.99	Down
M348T557_1	Adenosine 3'-monophosphate	157442454.99	125383224.52	0.80	-0.33	0.0074	2.23	Down
M352T531	Adenosine 2',3'-cyclic monophosphate	5012582.65	2494024.66	0.50	-1.01	0.0053	2.24	Down
M427T647	L-cysteine-glutathione disulfide	59867378.92	21004506.14	0.35	-1.51	0.0144	2.06	Down
M428T331	Stearoylcarnitine	80860853.27	48402600.94	0.60	-0.74	0.0143	2.08	Down
M440T352	Arvanil	4823689.50	2191750.20	0.45	-1.14	0.0153	2.06	Down
M482T352	1-hexadecyl-sn-glycero-3-phosphocholine	30320633.57	15653806.73	0.52	-0.95	0.0371	1.89	Down
M496T348	1-palmitoyl-sn-glycero-3-phosphocholine	1304272324.46	816962560.27	0.63	-0.67	0.0474	1.80	Down
M510T347	1-heptadecanoyl-sn-glycero-3-phosphocholine	75890617.61	61358175.78	0.81	-0.31	0.0065	2.26	Down
M524T345	1-Stearoyl-sn-glycerol 3-phosphocholine(LPC(18:0))	1916521371.65	1052748390.56	0.55	-0.86	0.0119	2.20	Down
M524T370	1-Stearoyl-2-hydroxy-sn-glycero-3-phosphocholine	33230704.76	23405876.91	0.70	-0.51	0.0403	1.83	Down

Supplementary Table S1: Analysis of differential metabolites between the STG and RTG groups in positive ion mode (continued).

ID	Name	Mean-STG	Mean-RTG	FC	log_2FC	*P*-value	VIP	Regulation
M684T637	Maltotetraose	15960387.02	6640412.40	0.42	-1.27	0.0451	1.73	Down
M751T318	1-palmitoyl-2-thiopalmitoyl phosphatidylcholine	4515572.91	2718988.61	0.60	-0.73	0.0185	2.00	Down
