# Supplementary material for: Allium mongolicum Regel-Mediated Rumen Microbiota Intervention Modulates Hepatic Metabolome to Reduce 4-Alkyl Branched-Chain Fatty Acids in Lamb Longissimus Thoracis Muscle
Source: Foods. 2026 May 7;15(10):1617. doi: 10.3390/foods15101617 (PMC13206602; doi:10.3390/foods15101617)
Supplement: Supplementary file 1 [file foods-15-01617-s001.zip › Supplementary Table S2.pdf]

Supplementary Table S2: Analysis of differential metabolites between the STG and AMG groups in positive ion mode.

ID	Name	Mean-STG	Mean-AMG	FC	$\log_2$FC	*P*-value	VIP	Regulation
M100T63	2-piperidone	35203031.21	97057117.62	2.76	1.46	0.0324	1.99	Up
M1032T325	Taurocholic acid	62908528.18	80202033.50	1.27	0.35	0.0234	2.02	Up
M107T408	Methyl 4,6-o-benzylidene-.alpha.-d-glucopyranoside	40880491.25	49236470.78	1.20	0.27	0.0088	2.22	Up
M110T58	Nicotinyl	29261728.86	54389146.93	1.86	0.89	0.0235	1.97	Up
M116T447_5	DL-arginine	7179474987.68	8277261816.53	1.15	0.21	0.0319	1.94	Up
M118T442_1	DL-Norvaline	3588211708.00	4261872942.95	1.19	0.25	0.0280	2.01	Up
M132T410_4	Leucine	10543719849.64	12188460032.31	1.16	0.21	0.0461	1.89	Up
M144T408	Met-His	182532962.95	220511575.16	1.21	0.27	0.0352	1.93	Up
M146T412	Oxyquinoline	53568512.57	66612973.63	1.24	0.31	0.0188	2.05	Up
M147T671_2	Lysine	1622434778.11	2066600426.06	1.27	0.35	0.0122	2.11	Up
M148T408	4,4'-bis(dimethylamino)benzophenone	10018412.89	12239985.84	1.22	0.29	0.0157	2.09	Up
M148T43	Formylanthranilic acid	51759997.62	74942407.12	1.45	0.53	0.0025	2.45	Up
M150T336	Synephrine	18098135.52	84635478.49	4.68	2.23	0.0083	2.36	Up
M164T497	Iberin	592180856.50	782656547.79	1.32	0.40	0.0290	1.95	Up
M166T408_4	Phenylalanine	6125036408.68	7407274514.03	1.21	0.27	0.0174	2.13	Up
M187T504	Ala-Pro	15143601.40	33284249.84	2.20	1.14	0.0432	1.90	Up
M204T435	N,n'-diacetylchitobiose	7695995.40	9652594.99	1.25	0.33	0.0496	1.84	Up
M221T161	Tyr-Cys-Arg	11280762.67	18718047.47	1.66	0.73	0.0149	2.02	Up
M243T80	Butylated hydroxytoluene	62751751.93	118047871.28	1.88	0.91	0.0410	1.81	Up
M258T524_3	Glycerophosphocholine	22807337151.15	25735109060.91	1.13	0.17	0.0384	1.83	Up
M275T351	Gigantol	29511029.56	45679766.87	1.55	0.63	0.0226	1.90	Up
M282T460	1-methyladenosine	32731716.25	38236723.04	1.17	0.22	0.0441	1.80	Up
M298T334	1-methylguanosine	22308953.19	28037817.57	1.26	0.33	0.0356	1.88	Up
M357T663	Arachidonyl trifluoromethyl ketone	9796761.44	22692064.17	2.32	1.21	0.0136	2.09	Up
M599T390	Phe-met-arg-phe-amide	1508576.61	3942574.15	2.61	1.39	0.0368	1.96	Up
M72T442	1,2-diamino-2-methylpropane	299661419.05	363652453.03	1.21	0.28	0.0345	1.96	Up
M86T410	Piperidine	1167747387.43	1345387470.79	1.15	0.20	0.0485	1.82	Up
M97T276	4-hydroxyhexenal	16885575.29	21246177.86	1.26	0.33	0.0266	1.97	Up
M324T604	Cytidine 5'-monophosphate	9351397.61	5573018.77	0.60	-0.75	0.0293	1.97	Down
M496T348	1-palmitoyl-sn-glycero-3-phosphocholine	1304272324.46	786263940.61	0.60	-0.73	0.0352	1.94	Down
M524T345	1-Stearoyl-sn-glycerol 3-phosphocholine(LPC(18:0))	1916521371.65	985977429.37	0.51	-0.96	0.0067	2.29	Down
M524T370	1-Stearoyl-2-hydroxy-sn-glycero-3-phosphocholine	33230704.76	21087508.92	0.63	-0.66	0.0091	2.14	Down
