# Supplementary material for: Allium mongolicum Regel-Mediated Rumen Microbiota Intervention Modulates Hepatic Metabolome to Reduce 4-Alkyl Branched-Chain Fatty Acids in Lamb Longissimus Thoracis Muscle
Source: Foods. 2026 May 7;15(10):1617. doi: 10.3390/foods15101617 (PMC13206602; doi:10.3390/foods15101617)
Supplement: Supplementary file 1 [file foods-15-01617-s001.zip › Supplementary Table S3.pdf]

Supplementary Table S3: Analysis of differential metabolites between the AMG and RTG groups in positive ion mode.

ID	Name	Mean-AMG	Mean-RTG	FC	log_2FC	*P*-value	VIP	Regulation
M158T397	2-(4-amino-1-piperidinyl)acetamide	3262366.00	3595395.58	1.10	0.14	0.0399	1.85	Up
M242T29	D-glucosamine, 6-sulfate	1431845.22	3024294.28	2.11	1.08	0.0183	2.04	Up
M344T78	Stearamide	2904134.77	4504595.40	1.55	0.63	0.0305	1.96	Up
M795T298	1-(1z-octadecenyl)-2-(5z,8z,11z,14z-eicosatetraenoyl)-sn-glycero-3-phosphocholine	5340715.08	9137706.50	1.71	0.77	0.0331	1.89	Up
M102T55	L-.alpha.-Amino-.gamma.-butyrolactone	68152059.04	36138461.21	0.53	-0.92	0.0200	2.04	Down
M110T58	Nicotinyl	54389146.93	33342148.99	0.61	-0.71	0.0413	1.84	Down
M150T336	Synephrine	84635478.49	25814942.97	0.31	-1.71	0.0270	2.10	Down
M164T497	Iberin	782656547.79	629756418.44	0.80	-0.31	0.0315	1.83	Down
M191T89	Thr-Ala	279892422.13	150519178.15	0.54	-0.89	0.0404	1.85	Down
M223T386	Phe-gly	157800205.14	79148543.32	0.50	-1.00	0.0352	1.87	Down
M243T80	Butylated hydroxytoluene	118047871.28	58898122.54	0.50	-1.00	0.0374	1.85	Down
M276T552	L-glutarylcarnitine	170326884.55	114928636.16	0.67	-0.57	0.0104	2.27	Down
M302T540	Tyr-Arg	14081668.92	4229947.02	0.30	-1.74	0.0042	2.37	Down
M352T531	Adenosine 2',3'-cyclic monophosphate	4847837.98	2494024.66	0.51	-0.96	0.0053	2.43	Down
M807T303	1-palmitoyl-2-docosahexaenoyl-sn-glycero-3-phosphocholine	222840664.50	167371422.31	0.75	-0.41	0.0390	1.88	Down
