# Supplementary material for: Allium mongolicum Regel-Mediated Rumen Microbiota Intervention Modulates Hepatic Metabolome to Reduce 4-Alkyl Branched-Chain Fatty Acids in Lamb Longissimus Thoracis Muscle
Source: Foods. 2026 May 7;15(10):1617. doi: 10.3390/foods15101617 (PMC13206602; doi:10.3390/foods15101617)
Supplement: Supplementary file 1 [file foods-15-01617-s001.zip › Supplementary Table S4.pdf]

Supplementary Table S4: Analysis of differential metabolites between the STG and AMG groups in negative ion mode.

ID	Name	Mean-STG	Mean-AMG	FC	log_2FC	*P*-value	VIP	Regulation
M101T54	Isovaleric acid	66824217.30	91109110.90	1.36	0.45	0.0445	1.73	Up
M104T520_3	DL-serine	424066345.23	542994570.68	1.28	0.36	0.0248	2.01	Up
M108T43	4-aminosalicylic acid	18303844.23	23567799.69	1.29	0.36	0.0265	1.96	Up
M114T447_4	DL-proline	901362367.35	1114075678.29	1.24	0.31	0.0157	2.19	Up
M116T443_6	Indole	2336367561.44	2832562243.31	1.21	0.28	0.0413	1.92	Up
M130T412_3	Norleucine	6056316294.37	7182756530.72	1.19	0.25	0.0390	1.93	Up
M131T243	Glutaric acid	2447179254.74	3518709220.70	1.44	0.52	0.0496	1.87	Up
M146T426	N-acetyl-dl-serine	14466688.68	28284624.32	1.96	0.97	0.0497	1.90	Up
M147T408_2	Phenyllactic acid	62693760.57	81482364.60	1.30	0.38	0.0166	2.20	Up
M148T431	L-methionine	339666773.79	445549325.55	1.31	0.39	0.0500	1.90	Up
M149T430	P-coumaryl alcohol	287192446.86	354206419.66	1.23	0.30	0.0254	1.96	Up
M158T321_2	Isovalerylglycine	502831652.41	860738772.64	1.71	0.78	0.0186	1.96	Up
M160T59	Indole-3-carboxylic acid	10013253.02	12108716.82	1.21	0.27	0.0138	2.10	Up
M164T408_3	DL-phenylalanine	2579199659.16	3290751873.71	1.28	0.35	0.0178	2.20	Up
M173T25	Isocitric acid	60282198.24	108230976.08	1.80	0.84	0.0152	2.03	Up
M176T408	2-Oxoadipic acid	6454646.83	14319476.26	2.22	1.15	0.0034	2.43	Up
M182T43_2	4-pyridoxic acid	1478548839.37	1874479501.06	1.27	0.34	0.0188	2.09	Up
M188T320	Asn-Gly	70551170.08	100403674.35	1.42	0.51	0.0244	1.98	Up
M201T25	Bergaptol	25252947.65	37536018.01	1.49	0.57	0.0416	1.79	Up
M203T412	Tryptophan	270940431.04	332736028.31	1.23	0.30	0.0486	1.89	Up
M207T620	Dl-lanthionine	5018743.35	8059352.74	1.61	0.68	0.0321	1.94	Up
M211T559	Benzoic acid, 3,4,5-trimethoxy-	4837948.68	5728630.71	1.18	0.24	0.0415	1.82	Up
M221T337	Aminomethylphosphonic acid	40951243.65	55888246.46	1.36	0.45	0.0409	1.85	Up
M236T299	Sepiapterin	173179026.84	250054328.22	1.44	0.53	0.0118	2.13	Up
M249T35	4,4'-sulfonylbisphenol	13247172.42	22041273.12	1.66	0.73	0.0239	1.91	Up
M263T43_1	3-methoxy-4-hydroxyphenylglycol sulfate	14321703.37	20273340.46	1.42	0.50	0.0007	2.51	Up
M269T44_2	Heptadecanoic acid	350331820.43	437361856.77	1.25	0.32	0.0382	1.78	Up
M281T183	4',7-dimethoxyisoflavone	5587971.22	15340056.28	2.75	1.46	0.0352	1.80	Up
M315T323	L-dihydroorotate	2096332.06	4205039.97	2.01	1.00	0.0498	1.82	Up
M74T504_4	Glycine	827856904.25	912698949.84	1.10	0.14	0.0243	1.90	Up
M89T337_5	L-(+)-lactic acid	41600637094.67	54822978042.17	1.32	0.40	0.0301	1.89	Up
M125T34	Ethyl sulfate	122958462.08	45626011.33	0.37	-1.43	0.0204	1.97	Down
M214T27	2-benzothiazolsulfonic acid	5001231.77	2351119.90	0.47	-1.09	0.0346	1.93	Down
M215T118	2-fluoro-4'-hydroxybenzophenone	157291705.73	90448713.62	0.58	-0.80	0.0340	1.86	Down
M227T524	3,5-dinitrosalicylate	7294374.53	3976995.61	0.55	-0.88	0.0361	1.80	Down
M244T52	Glyphosine	28307950.43	16219242.05	0.57	-0.80	0.0275	1.94	Down
M251T225	2'-methoxyflavone	52444892.06	24712015.80	0.47	-1.09	0.0211	1.95	Down
M257T216	Tetradecanedioic acid	19811265.88	8609221.66	0.43	-1.20	0.0059	2.19	Down
M329T55	11beta-hydroxyprogesterone	20836627.64	5830873.23	0.28	-1.84	0.0168	2.06	Down
