# Supplementary material for: Allium mongolicum Regel-Mediated Rumen Microbiota Intervention Modulates Hepatic Metabolome to Reduce 4-Alkyl Branched-Chain Fatty Acids in Lamb Longissimus Thoracis Muscle
Source: Foods. 2026 May 7;15(10):1617. doi: 10.3390/foods15101617 (PMC13206602; doi:10.3390/foods15101617)
Supplement: Supplementary file 1 [file foods-15-01617-s001.zip › Supplementary Table S5.pdf]

Supplementary Table S5: Analysis of differential metabolites between the STG and RTG groups in negative ion mode.

ID	Name	Mean-STG	Mean-RTG	FC	log_2FC	*P*-value	VIP	Regulation
M101T54	Isovaleric acid	66824217.30	106347019.79	1.59	0.67	0.0031	2.42	Up
M102T25_2	D-2-aminobutyrate	5555531.24	10484447.77	1.89	0.92	0.0369	1.83	Up
M108T43	4-aminosalicylic acid	18303844.23	23872333.26	1.30	0.38	0.0409	1.86	Up
M114T447_4	DL-proline	901362367.35	1080624715.43	1.20	0.26	0.0188	2.15	Up
M130T412_3	Norleucine	6056316294.37	6765507037.35	1.12	0.16	0.0019	2.44	Up
M141T41	2-mercaptoethanesulfonic acid	107567173.49	198994081.64	1.85	0.89	0.0499	1.81	Up
M144T340	Isobutyrylglycine	50346935.04	108516096.61	2.16	1.11	0.0097	2.20	Up
M144T44	Quinolin-2-ol	14077288.42	21614602.31	1.54	0.62	0.0317	1.92	Up
M146T426	N-acetyl-dl-serine	14466688.68	18975510.22	1.31	0.39	0.0441	1.85	Up
M147T408_2	Phenyllactic acid	62693760.57	74494392.75	1.19	0.25	0.0024	2.55	Up
M148T431	L-methionine	339666773.79	408223694.59	1.20	0.27	0.0031	2.48	Up
M151T338_2	Oxypurinol	3302826906.95	4449802411.60	1.35	0.43	0.0492	1.81	Up
M152T525	L-Cysteinesulfinic acid	18200265.66	24725508.46	1.36	0.44	0.0015	2.55	Up
M153T388	Benzylphosphonic acid	68135928.71	104104094.58	1.53	0.61	0.0006	2.54	Up
M158T321_2	Isovalerylglycine	502831652.41	980571764.37	1.95	0.96	0.0067	2.28	Up
M160T556	2-aminoadipic acid	16032183.66	20242569.62	1.26	0.34	0.0304	1.90	Up
M164T408_3	DL-phenylalanine	2579199659.16	3022697001.56	1.17	0.23	0.0050	2.48	Up
M168T507	L-Cysteic acid	9308286.22	12179628.99	1.31	0.39	0.0034	2.46	Up
M173T25	Isocitric acid	60282198.24	244318688.31	4.05	2.02	0.0114	2.22	Up
M175T516	Serotonin	10972881.26	14039240.16	1.28	0.36	0.0242	2.06	Up
M179T31	Caffeic acid	3471447.05	9140997.45	2.63	1.40	0.0103	2.25	Up
M182T43_2	4-pyridoxic acid	1478548839.37	1896012883.84	1.28	0.36	0.0152	2.18	Up
M200T440	Cysteine-s-sulfate	4950238.32	8908847.71	1.80	0.85	0.0068	2.27	Up
M203T412	Tryptophan	270940431.04	319958253.00	1.18	0.24	0.0027	2.45	Up
M205T487	Thr-Ser	27766507.06	41479378.33	1.49	0.58	0.0146	2.11	Up
M207T620	Dl-lanthionine	5018743.35	8615376.33	1.72	0.78	0.0410	1.84	Up
M217T415	Val-Thr	54329721.19	102658387.31	1.89	0.92	0.0072	2.27	Up
M218T527	Ser-Asn	3963155.99	7288234.68	1.84	0.88	0.0038	2.34	Up
M231T392	Ile-Thr	9597570.89	16293246.18	1.70	0.76	0.0095	2.25	Up
M232T518	Ser-Gln	2928556.49	4758975.88	1.63	0.70	0.0162	2.07	Up
M244T447	Ile-Asn	8196091.77	15714980.38	1.92	0.94	0.0080	2.23	Up
M258T440	2-heptyl-4-hydroxyquinoline n-oxide	4567799.57	8956750.30	1.96	0.97	0.0498	1.78	Up
M259T537	Norfloxacin	2160813.38	3700914.83	1.71	0.78	0.0186	2.07	Up
M260T434	O-phosphotyrosine	4037320.99	5490472.05	1.36	0.44	0.0123	2.17	Up
M279T75	Chaulmoogric acid	155315572.23	475381837.32	3.06	1.61	0.0300	1.87	Up
M281T183	4',7-dimethoxyisoflavone	5587971.22	13454018.28	2.41	1.27	0.0210	2.04	Up
M74T504_4	Glycine	827856904.25	908161975.46	1.10	0.13	0.0071	2.27	Up
M125T34	Ethyl sulfate	122958462.08	31757335.92	0.26	-1.95	0.0035	2.42	Down
M127T131	Dihydro-4,4-dimethyl-2,3-furandione	22564903.48	16358371.06	0.72	-0.46	0.0422	1.85	Down
M143T72	Chlorohydroquinone	409740360.25	272834446.98	0.67	-0.59	0.0491	1.84	Down
M159T71_2	2,2-dimethylglutaric acid	28576842.43	15184227.19	0.53	-0.91	0.0246	1.96	Down
M162T317	Pterine	29297270.81	18887447.33	0.64	-0.63	0.0311	1.96	Down
M171T1	L-homocitrulline	9067552.75	5293199.29	0.58	-0.78	0.0411	1.83	Down
M237T247	2-keto-3-deoxyoctonic acid	47922763.82	23977022.21	0.50	-1.00	0.0411	1.81	Down
M255T44_4	Hexadecanoic acid	4947365352.54	3889691110.86	0.79	-0.35	0.0482	1.78	Down
M274T579	Gamma-glu-glu	27231711.77	14369119.33	0.53	-0.92	0.0357	1.96	Down
M303T319	3,3',4,5'-Tetrahydroxy-trans-stilbene	25738745.47	11414477.94	0.44	-1.17	0.0011	2.47	Down
M346T556	Adenosine 2'-monophosphate	54065964.98	45319870.34	0.84	-0.25	0.0048	2.36	Down
