# Supplementary material for: Allium mongolicum Regel-Mediated Rumen Microbiota Intervention Modulates Hepatic Metabolome to Reduce 4-Alkyl Branched-Chain Fatty Acids in Lamb Longissimus Thoracis Muscle
Source: Foods. 2026 May 7;15(10):1617. doi: 10.3390/foods15101617 (PMC13206602; doi:10.3390/foods15101617)
Supplement: Supplementary file 1 [file foods-15-01617-s001.zip › Supplementary Table S6.pdf]

Supplementary Table S6: Analysis of differential metabolites between the AMG and AMG groups in negative ion mode.

ID	Name	Mean-AMG	Mean-RTG	FC	log_2FC	*P*-value	VIP	Regulation
M152T525	L-Cysteinesulfinic acid	19257832.57	24725508.46	1.28	0.36	0.0167	2.14	Up
M168T507	L-Cysteic acid	9799353.98	12179628.99	1.24	0.31	0.0181	2.10	Up
M215T118	2-fluoro-4'-hydroxybenzophenone	90448713.62	229507224.53	2.54	1.34	0.0010	2.61	Up
M217T415	Val-Thr	59915227.78	102658387.31	1.71	0.78	0.0423	1.96	Up
M218T527	Ser-Asn	3856394.32	7288234.68	1.89	0.92	0.0048	2.38	Up
M239T576	.gamma.-aminobutyryl-l-histidine	29031943.34	59158127.31	2.04	1.03	0.0198	2.08	Up
M259T537	Norfloxacin	1961745.18	3700914.83	1.89	0.92	0.0104	2.24	Up
M260T434	O-phosphotyrosine	4310771.52	5490472.05	1.27	0.35	0.0090	2.30	Up
M116T443_5	Guanidoacetic acid	4445110.83	3758183.55	0.85	-0.24	0.0334	1.97	Down
M118T501_2	DL-threonine	965536163.26	825552953.18	0.86	-0.23	0.0328	1.94	Down
M127T131	Dihydro-4,4-dimethyl-2,3-furandione	27842257.70	16358371.06	0.59	-0.77	0.0092	2.26	Down
M145T161	1-(2-hydroxyphenyl)-3-phenyl-1,3-propanedione	162717523.26	72013571.21	0.44	-1.18	0.0372	1.97	Down
M157T91	2-oxooctanoic acid	23067673.71	14820669.29	0.64	-0.64	0.0425	1.86	Down
M176T408	2-Oxoadipic acid	14319476.26	8215286.19	0.57	-0.80	0.0257	2.00	Down
M188T320	Asn-Gly	100403674.35	73171147.84	0.73	-0.46	0.0448	1.85	Down
M195T28	4',6'-dimethoxy-2'-hydroxyacetophenone	8033571.92	4148394.63	0.52	-0.95	0.0487	1.86	Down
M202T300	Indole-3-butyric acid	552546906.49	420921213.47	0.76	-0.39	0.0301	1.95	Down
M209T28	N-octyl sulfate	56703213.66	23905265.93	0.42	-1.25	0.0329	1.91	Down
M215T407	D-psicose	27942542.34	19361562.70	0.69	-0.53	0.0151	2.18	Down
M223T29_2	2-hydroxyanthraquinone	98746815.00	39100200.52	0.40	-1.34	0.0490	1.81	Down
M227T44	Myristic acid	718999972.44	401781848.12	0.56	-0.84	0.0293	1.99	Down
M228T517	N-tris(hydroxymethyl)methyl-2-aminoethanesulfonic acid	26234267.98	14073141.59	0.54	-0.90	0.0007	2.62	Down
M236T299	Sepiapterin	250054328.22	159336245.25	0.64	-0.65	0.0039	2.46	Down
M241T44	Pentadecanoic acid	387494595.60	219859714.46	0.57	-0.82	0.0195	2.12	Down
M247T30	Fludioxonil	12671217.16	6700192.87	0.53	-0.92	0.0158	2.22	Down
M263T43_1	3-methoxy-4-hydroxyphenylglycol sulfate	20273340.46	15300251.35	0.75	-0.41	0.0163	2.22	Down
M269T44_2	Heptadecanoic acid	437361856.77	312002309.35	0.71	-0.49	0.0112	2.23	Down
M287T45	Testosterone	11985911.92	8398401.42	0.70	-0.51	0.0245	2.08	Down
M303T319	3,3',4,5'-Tetrahydroxy-trans-stilbene	27675663.42	11414477.94	0.41	-1.28	0.0018	2.53	Down
