# Supplementary material for: Allium mongolicum Regel-Mediated Rumen Microbiota Intervention Modulates Hepatic Metabolome to Reduce 4-Alkyl Branched-Chain Fatty Acids in Lamb Longissimus Thoracis Muscle
Source: Foods. 2026 May 7;15(10):1617. doi: 10.3390/foods15101617 (PMC13206602; doi:10.3390/foods15101617)
Supplement: Supplementary file 1 [file foods-15-01617-s001.zip › Supplementary Table S7.pdf]

Supplementary Table S7: KEGG enrichment analysis of the top 20 nominal differential metabolites.

PathwayID	Pathway	Level1	Level2	*P*-value	FDR
map04974	Protein digestion and absorption	Organismal Systems	Digestive system	<0.001	<0.001
map01230	Biosynthesis of amino acids	Metabolism	Global and overview maps	<0.001	<0.001
map04978	Mineral absorption	Organismal Systems	Digestive system	<0.001	<0.001
map00970	Aminoacyl-tRNA biosynthesis	Genetic Information Processing	Translation	<0.001	<0.001
map00470	D-Amino acid metabolism	Metabolism	Metabolism of other amino acids	<0.001	<0.001
map02010	ABC transporters	Environmental Information Processing	Membrane transport	<0.001	<0.001
map01210	2-Oxocarboxylic acid metabolism	Metabolism	Global and overview maps	<0.001	<0.001
map00380	Tryptophan metabolism	Metabolism	Amino acid metabolism	<0.001	<0.001
map00270	Cysteine and methionine metabolism	Metabolism	Amino acid metabolism	<0.001	<0.001
map00310	Lysine degradation	Metabolism	Amino acid metabolism	<0.001	0.003
map04361	Axon regeneration	Organismal Systems	Development and regeneration	<0.001	0.004
map00300	Lysine biosynthesis	Metabolism	Amino acid metabolism	0.002	0.007
map00400	Phenylalanine, tyrosine and tryptophan biosynthesis	Metabolism	Amino acid metabolism	0.002	0.007
map04721	Synaptic vesicle cycle	Organismal Systems	Nervous system	0.003	0.011
map00260	Glycine, serine and threonine metabolism	Metabolism	Amino acid metabolism	0.004	0.014
map04080	Neuroactive ligand-receptor interaction	Environmental Information Processing	Signaling molecules and interaction	0.005	0.018
map00630	Glyoxylate and dicarboxylate metabolism	Metabolism	Carbohydrate metabolism	0.009	0.028
map00430	Taurine and hypotaurine metabolism	Metabolism	Metabolism of other amino acids	0.011	0.033
map01240	Biosynthesis of cofactors	Metabolism	Global and overview maps	0.021	0.059
map04150	mTOR signaling pathway	Environmental Information Processing	Signal transduction	0.027	0.072
